# A novel subject-wise dictionary learning approach using multi-subject fMRI spatial and temporal components

**DOI:** 10.1038/s41598-023-47420-1

**Published:** 2023-11-18

**Authors:** Muhammad Usman Khalid, Malik Muhammad Nauman

**Affiliations:** 1 College of Computer and Information Sciences, Imam Mohammad Ibn Saud Islamic University, 11564 Riyadh, Saudi Arabia; 2 Faculty of Integrated Technologies, Universiti Brunei Darussalam, Bandar Seri Begawan, BE1410 Brunei

**Keywords:** Electrical and electronic engineering, Imaging, Magnetic resonance imaging

## Abstract

The conventional dictionary learning (DL) algorithms aim to adapt the dictionary/sparse code to individual functional magnetic resonance imaging (fMRI) data. Thus, lacking the capability to consolidate the spatiotemporal diversities offered by other subjects. Considering that subject-wise (sw) data matrix can be decomposed into the sparse linear combination of multi-subject (MS) time courses and MS spatial maps, two new algorithms, sw sequential DL (swsDL) and sw block DL (swbDL), have been proposed. They are based on the novel framework, defined by the mixing model, where base matrices prepared by operating a computationally fast sparse spatiotemporal blind source separation method over multiple subjects are employed to adapt the mixing matrices to sw training data. They solve the optimization models formulated using $l_0$/$l_1$-norm penalization/constraints through dictionary/sparse code pair update and alternating minimization approach. They are unique because no existing sparse DL method can incorporate MS spatiotemporal components while updating sw atoms/sparse codes, which can eventually be assembled using neuroscience knowledge to extract group-level dynamics. Various fMRI datasets are used to evaluate and compare the performance of the proposed algorithms with existing state-of-the-art algorithms. Specifically, overall, a $14\%$ increase in the mean correlation value and $39\%$ reduction in the mean computation time exhibited by swsDL and swbDL, respectively, over the adaptive consistent sequential dictionary algorithm.

## Introduction

Due to its high spatial resolution, fMRI has emerged as an effective neuroimaging technique for capturing brain activity during rest or cognition^1^. Brain scans from fMRI are usually analyzed using the multivariate general linear model (GLM)^2^, which relies on the experimental paradigm and hemodynamic response function (HRF) for its design matrix^3^. Consequently, this approach becomes ineffective for the resting-state investigations where the dynamics of the experiment are hard to model and when HRF has variations across subjects^4^. For such complex scenarios, data-driven methods are preferred due to their reduced assumptions about the underlying structure of the data^5^. They can adapt to individual and regional hemodynamics across subjects and brain regions by learning underlying trends from the training data^6,7^. This makes them practical for both task-related activation detection and resting-state functional connectivity analysis. In this regard, blind source separation (BSS) based matrix decomposition methods that unveil hidden structures in the multivariate data have been very consequential for fMRI studies^8–16^.

Among the data-driven methods for brain imaging, spatial ICA (sICA) has enjoyed the most success^17^. This is due to its numerical simplicity and lower spatial variations of fMRI than temporal^18,19^. However, it has been suggested that independence is questionable for fMRI and that the sparsity of components is a more productive assumption^20^. A later study refuted this claim and found that both sparsity and independence are valid presumptions for fMRI analysis^21^. Moreover, ICA’s assumption of independence was once more questioned in^22,23^, where it was found that, in contrast to sparse DL, ICA had trouble retrieving neural dynamics when there were moderate to significant overlaps among functional networks. Biological evidence of sparse coding in the brain^24^, a previous ICA investigation^25^, and a more recent evidence of sparse brain networks^26^ have all supported the sparse assumption. Eventually, a more plausible framework, sparse spatial ICA (ssICA), that can jointly exploit both source diversities through step-wise optimization strategy was developed for fMRI^27^.

Since the advent of compressed sensing^28^, sparse representation^29^ has been extensively utilized to address various signal and image processing problems^30–35^. It is particularly fruitful for fMRI when combined with sequential/block dictionary learning^36^, which allows representing the blood-oxygen-level-dependent (BOLD) signal by means of a few dictionary atoms via sparse code. The data decomposition using sparse DL inspired authors in^37^ to create a pioneering sparse GLM framework for fMRI. Subsequently, numerous DL algorithms have since been created, especially for single-subject fMRI data^38–44^. However, none of these subject-wise dictionary models have the flexibility to accommodate neural variations and complexities across multiple subjects. Due to the statistical power of the multivariate analysis, incorporating MS-fMRI data into the subject-wise decomposition may enhance the estimation efficiency of each individual’s recovered TCs and SMs in terms of signal-to-noise ratio (SNR) and spatial sensitivity, respectively^45–47^.

Motivated by the recently presented approach that consisted of a common autoencoder that projected each subject’s fMRI data to shared embedding space followed by a subject-specific decoder that reconstructed data for each subject^47^, two new algorithms, swbDL and swsDL for subject-wise as well group-wise source retrieval are proposed in this paper. Instead of aggregating the dictionary atoms across subjects in a lower-dimensional space^48^, we propose that a reverse strategy would be much more optimal due to its ability to take advantage of the DL’s data reconstruction model. Considering the spatial and temporal components from multiple subjects as preliminary bases, the explicit dictionary/sparse code is obtained by training the representation matrices sequentially and block-wise. These spatiotemporal components were obtained using the ssBSS method^49^ that exploits concurrent feature extraction^48^ in a computationally efficient manner.

The proposed model differs from the existing models that consider constructing only the base dictionary using DCT transform^40,50^. Instead of using a single-cycle learning approach where DCT bases are used as a base dictionary to train the representation matrix, a double-cycle, and a double-representation matrix training is adopted. In the first cycle, quick explicit implementation is realized to train a base dictionary and the base sparse code, and in the second cycle, associated representation matrices are trained. This strategy ensures that both base atom/sparse code matrices span their respective signal space from multiple subjects.

## Related work

While some features and white noise in fMRI data vary significantly across subjects, responses to experimental stimuli and resting-state networks share common features that are jointly discoverable. To synergize the source signals recovery across multiple subjects, consider three aspects of the group fMRI data analysis (a) common TCs and corresponding common SMs are found only in task-related data, (b) unique TCs and corresponding common SMs are found both in task-related and resting-state data, and (c) unique TCs and corresponding unique SMs are found both in task-related and resting-state data.

Due to their model restricted to realize both (a) and (c) simultaneously by aggregating the common temporal dynamics across subjects, hybrid concatenation scheme (HCSDL)^51^, shared and subject-specific DL (ShSSDL)^52^, low-rank Tucker 2 model (LRT-2)^53^, sparse group bases (sgBACES)^50^, and sparse alternating rank-R/1 least squares (sRrR1LS)^54^ merely learn common and subject-specific TCs/SMs and therefore cannot handle resting-state datasets or produce subject-wise dynamics. On the other hand, multi-subject DL (MSDL)^55^ conceptualized only (b) and to some extent (c), resulting in its applicability to resting-state datasets only, and due to this, cannot accurately retrieve subject-wise responses.

For subject-wise analysis, the group-level method must be able to handle (c) independent of (a) or (b). The only such methods in the literature are group sICA^18^ and compressed online DL (CODL)^56^. They naturally retrieve subject-wise TCs/SMs through the population-level spatial maps. They are most versatile because they can entertain all three types of fMRI data analysis mentioned above; however, cgICA might yield inferior basis/maps when spatial dependence among underlying sources is significant, which can be resolved by replacing sICA with ssICA^27^. Whereas the DL-based methods such as supervised DL (SDL)^57^ and supervised stochastic coordinate coding (SCC)^58^ that consist of integrating the model and data-driven approach are capable of subject-wise learning, but they are unable to capture the diversity of brain activities and networks across subjects for TCs/SMs of interest.

Recently, some methods have been developed to exploit statistical dependencies across subjects to enhance the sensitivity of brain responses and reduce estimation error for each individual. For instance, a shared response model (SRM) was proposed by authors in^45^ to decompose each subject’s data into a subject-wise basis and a matrix of shared responses, a multi-subject low-ranked (LR) joint model, MS-LR-GLM, was proposed by authors in^46^ that enabled combining the data variations across subjects to enhance the subject-wise HRF estimation, and more recently, a non-linear model named MRMD-AE resulted in higher classification accuracy of stimulus relevant fMRI signal due to synergy between multiple subjects^47^.

To the best of my knowledge, no dictionary learning method in literature to date can exploit the shared responses across subjects to enhance the subject-wise analysis. The proposed algorithms in this paper fill this gap elegantly.

## Background

The ACSD algorithm^41^ had established that instead of relying on the conventional alternating minimization approach as proposed in the KSVD algorithm, updating elements of the dictionary and the corresponding sparse code jointly by solving a penalized rank one error matrix approximation that promotes an adaptive sparse penalty lead to faster convergence and overall higher atom recovery percentage.

**Figure 1:**
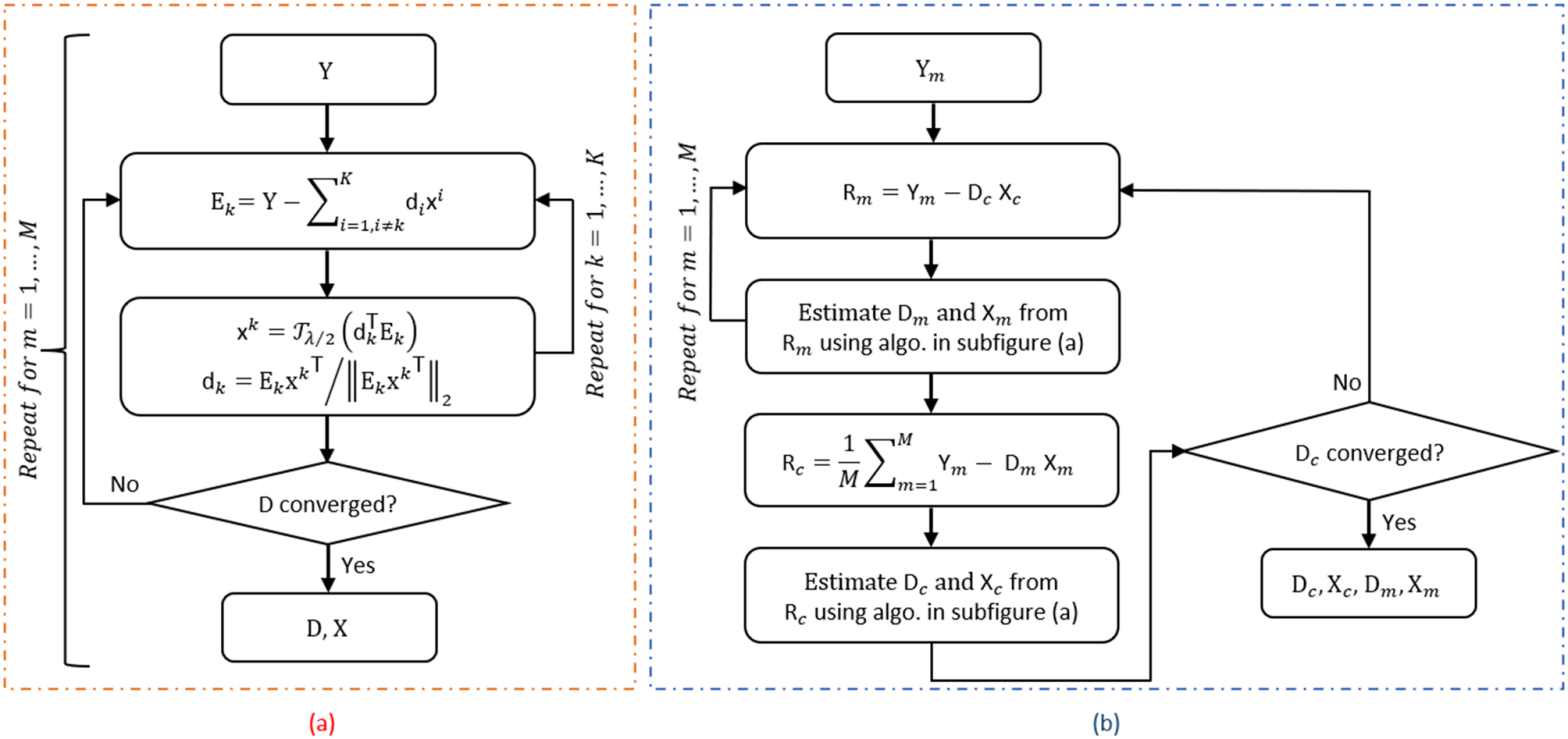
A flowchart describing the ACSD algorithm for (**a**) subject-wise analysis modeled by Eq. (1)^41^, and (**b**) multi-subject group analysis modeled by Eq. (2)^50^.

For this purpose, consider the fMRI data matrix $\text {Y} \in \mathbb {R}^{N\times V}$ constructed from whole-brain BOLD time series that consists of *N* scans and *V* voxels. Assuming there is sparseness along the row direction of the fMRI data matrix, then according to the ACSD approach, it can be decomposed into a dictionary matrix $\text {D} \in \mathbb {R}^{N\times K}$ whose columns have normalization constraint and the sparse code matrix $\text {X} \in \mathbb {R}^{K\times V}$ whose entries have adaptive sparse penalty expressed as

1 $$\begin{aligned}&\min _{\text {D}, \text {X}}\left\Vert \text {Y} -\text {D}\text {X}\right\Vert _F^2+\sum _{k=1}^{K}\sum _{j=1}^{V}\lambda _{j}^k|x_{j}^k|, \quad \text {sub.to.} \quad \left\Vert \text {d}_{k}\right\Vert _2 =1 \end{aligned}$$

where $\left\Vert .\right\Vert _{F}$ and $\left\Vert .\right\Vert _2$ is the Frobenius and $l_2$ norm, respectively, |.| represents the absolute value, hyperparameter $\lambda _j^k$ is the data-driven regularization parameter allocated to each entry of $\text {X}$, $N<K$ signifies overcomplete dictionary, and each atom is normalized to avoid scaling ambiguity. The ACSD algorithm aims to solve Eq. (1) by penalizing the coefficient row in the full error matrix based rank-1 minimization problem to promote the sparsity of $\text {x}^k$ as

$$\begin{aligned}{}&\{\text {d}_{k},\text {x}^k\}=\arg \min _{\text {d}_k, \text {x}^k} \left\Vert \text {E}_{k} -\text {d}_{k}\text {x}^k\right\Vert _{F}^2+\sum _{j=1}^{V}\lambda _{j}^k|x_{j}^k|, \quad \text {sub.to.} \quad \left\Vert \text {d}_{k}\right\Vert _2 =1 \end{aligned}$$

where $\text {E}_{k} = \text {Y}-\sum _{i=1,i\ne k}^{K}\text {d}_{i}\text {x}^i$ is the error matrix for all signals from which the *k*-th atom/sparse code has been removed. The resulting estimate of $\text {x}^k/\text {d}_{k}$ as a pair is given by

$$\begin{aligned}{}&{\text {x}^k}= \mathcal {T}_{\lambda /2}\Big (\text {d}_k^\top \text {E}_{k}\Big )\\&{\text {d}_{k}}= \text {E}_{k} {\text {x}^k}^\top \Big /\left\Vert \text {E}_{k} {\text {x}^k}^\top \right\Vert _2 \end{aligned}$$

where $\mathcal {T}_\upsilon (\text {z})=\textrm{sgn}(\text {z})\circ (|\text {z}|-\upsilon 1/|\text {z}|)_+$, $(\textrm{z})_+$, $\textrm{sgn}(.)$, and $\circ$ define the component-wise max between $(0,\textrm{z})$, the component-wise sign, and the Hadamard product, respectively^59^, 1 is a vector of ones, and $\varvec{\lambda }^k=[\lambda ^k_1,\ldots ,\lambda ^k_V]$ is obtained using single tuning parameter as $\lambda 1/|\text {d}_k^\top \text {E}_{k}|$. Figure 1a describes these updates in form of a flow chart. For fMRI group analysis Eq. (1) is modified according to the sgBACES algorithm^50^ as

2 $$\begin{aligned}{}&\min _{\text {D}_c,\text {X}_c,\text {D}_m,\text {X}_m}\sum _{m=1}^{M}\left\Vert \text {Y}_m -\text {D}_c\text {X}_c-\text {D}_m\text {X}_m\right\Vert _{F}^2+\sum _{k=1}^{K}\sum _{j=1}^{V}\zeta ^k_{c,j}|x^k_{c,j}|+\sum _{k=1}^{K}\sum _{j=1}^{V}\zeta ^k_{m,j}|x^k_{m,j}|, \quad \text {sub.to.} \ \ \left\Vert \text {d}_{c,k}\right\Vert _2 =1, \ \ \left\Vert \text {d}_{m,k}\right\Vert _2 =1 \end{aligned}$$

where $m=\{1,\ldots ,M\}$, *M* is the number of subjects, $\text {D}_c/\text {X}_c$ stand for the common dictionary/sparse code, and $\text {D}_m/\text {X}_m$ represent the subject-specific dictionary/sparse code. The *k*-th atom/sparse code update for the subject-specific dynamics is obtained by considering the subject-level residuals $\text {R}_m=\text {Y}_m-\text {D}_c\text {X}_c$ and error matrix for all signals $\text {E}_{m,k} = \text {R}_m-\sum _{i=1,i\ne k}^{K_m}\text {d}_{m,i}\text {x}_m^i$ as

$$\begin{aligned}{}&\{\text {d}_{m,k},\text {x}_m^k\}=\arg \min _{\text {d}_{m,k}, \text {x}_m^{k}}\left\Vert \text {E}_{m,k}-\text {d}_{m,k}\text {x}_m^k\right\Vert _{F}^2 +\sum _{j=1}^{V}\zeta _{m,j}^k|x_{m,j}^k|, \quad \text {sub.to.} \ \ \left\Vert \text {d}_{m,k}\right\Vert _2 =1 \end{aligned}$$

Whereas the *k*-th atom/sparse code update for the subject-specific dynamics is obtained by considering the common-level residuals $\text {R}_c=1/M\sum _{m=1}^{M}\text {Y}_m-\text {D}_m\text {X}_m$ and error matrix $\text {E}_{c,k} = \text {R}_c-\sum _{i=1,i\ne k}^{K_c}\text {d}_{c,i}\text {x}_c^i$ as

$$\begin{aligned}{}&\{\text {d}_{c,k},\text {x}_c^k\}=\arg \min _{\text {d}_{c,k}, \text {x}_c^{k}}\left\Vert \text {E}_{c,k}-\text {d}_{c,k}\text {x}_c^k\right\Vert _{F}^2+\sum _{j=1}^{V}\zeta _{c,j}^k|x_{c,j}^k|, \quad \text {sub.to.} \ \ \left\Vert \text {d}_{c,k}\right\Vert _2 =1. \end{aligned}$$

The atom/sparse code row in the two equations mentioned above can be solved sequentially as a pair using the ACSD algorithm. Figure 1b gives a flow chart of this routine.

## Methods

The DL algorithms, ACSD in particular, have shown superior convergence properties and source recovery precision compared to other BSS methods. Despite this, their applicability is limited to either conventional single-subject analysis^41^ or multi-subject based group analysis^50^ as shown in Fig. 1. This paper mainly focuses on extending the DL algorithm to multi-subject based subject-wise analysis to enhance the statistical strength of the single-subject analysis. This expansion would strengthen the accuracy of subject-wise analysis by exploiting hemodynamic variations offered by multiple subjects. It is worth mentioning at this point that sgBACES^50^ can also be extended to subject-wise analysis by not combining the common-level residuals and performing DL on each of those residuals individually; however, this approach will raise the computational complexity and remain inapplicable to resting-state data that requires shared spatial maps.

### Proposed model

The proposed algorithms secure the swiftly extracted underlying spatial/temporal components from multiple subjects using the ssBSS method and adapt them to subject-wise analysis resulting in increased source recovery performance for each individual. In this regard, consider that each signal in fMRI dataset $\text {Y}_m \in \mathbb {R}^{N\times V}$ from *m*-th subject can be represented as a linear combination of a few atoms from subject-wise dictionary $\text {D}_m \in \mathbb {R}^{N\times K}$ according to the sparse signal strength in each column of the subject-wise coefficient matrix $\text {X}_m \in \mathbb {R}^{K\times V}$. However, these subject-wise matrices are constructed using multi-subject atoms/sparse code, which can be accounted for by the multi-subject smooth dictionary $\text {D}_q \in \mathbb {R}^{N\times MP}$ and the multi-subject sparse code $\text {X}_q \in \mathbb {R}^{MP\times V}$ treated as base dictionary and base sparse code, respectively. This leads to $\text {D}_m=\text {D}_q\text {A}_m$ and $\text {X}_m=\text {B}_m\text {X}_q$. The proposed model in its basic form is given as

3 $$\begin{aligned}{}&\min _{\text {A}_m, \text {B}_m}\left\Vert \text {Y}_m -\text {D}_q\text {A}_m\text {B}_m\text {X}_q\right\Vert _F^2+\lambda \left\Vert \text {b}_m^k\text {X}_q\right\Vert _1, \quad \text {sub.to.}\ \ \left\Vert \text {D}_q\text {a}_{m,k}\right\Vert _2 =1 \end{aligned}$$

where $\text {A}_m \in \mathbb {R}^{MP\times K}$ and $\text {B}_m \in \mathbb {R}^{K\times MP}$ are the representation matrices, $\text {a}_{m,k}$ and $\text {b}_m^k$ are the *k*-th column of $\text {A}_m$ and *k*-th row of $\text {B}_m$, respectively, $K<N<MP<V$, and *M* is the number of subjects. The next subsection describes how to quickly train $\text {D}_q$ and $\text {X}_q$.

### Proposed preliminaries

The ssBSS method^49^ proposed the following optimization model by considering that dataset $\text {Y}_m$ can be decomposed into temporal source matrix $\text {T}_m \in \mathbb {R}^{N\times P}$ and spatial source matrix $\text {S}_m \in \mathbb {R}^{P\times V}$ as

4 $$\begin{aligned} \min _{\text {C}_m, \text {S}_m}\left\Vert \text {Y}_m -\text {T}_p\text {C}_m\text {S}_m\right\Vert _F^2+\lambda _1\left\Vert \text {S}_m\right\Vert _1, \quad \text {sub.to.} \ \ \left\Vert \text {T}_p\text {c}_{m,p}\right\Vert _2 =1, \quad \left\Vert \text {c}_{m,p}\right\Vert _0\le \zeta _1 \end{aligned}$$

where $\text {T}_m=\text {T}_p\text {C}_m$ accounts for the smoothness of the BOLD signal by storing DCT bases in $\text {T}_p \in \mathbb {R}^{N\times K_p}$, and $\text {c}_{m,p}$ is the *p*-th column of the sparse representation matrix $\text {C}_m$, $P<K_p<N$. $\left\Vert .\right\Vert _0$ is the $l_0$ norm that induces sparsity by counting the number of non-zero elements, $\left\Vert \text {S}_m\right\Vert _1$ is the $l_1$ norm on $\text {S}_m$ given as $\sum _{k=1}^{K} \sum _{j=1}^{V}|s^k_{m,j}|$, and $\lambda _1$ is the sparsity hyperparameter that regulates the coefficient values. To solve Eq. (4) efficiently, blind source separation theory is employed that breaks it into the following pair of spatial and temporal source separation problems

$$\begin{aligned}{}&\min _{\text {C}_m, \text {Q}_m}\left\Vert \text {X}_{m,t} -\text {T}_p\text {C}_m\text {Q}_m\right\Vert _F^2+\lambda _1\left\Vert \text {Q}_m\right\Vert _1, \quad \text {sub.to.} \ \ \left\Vert \text {T}_p\text {c}_{m,p}\right\Vert _2 =1, \quad \left\Vert \text {c}_{m,p}\right\Vert _0\le \zeta _1 \\&\min _{\text {S}_m, \text {Z}_m}\left\Vert \text {X}_{m,s} -\text {Z}_m\text {S}_m\right\Vert _F^2+\lambda _2\left\Vert \text {Z}_m\right\Vert _1+\lambda _3\left\Vert \text {S}_m\right\Vert _1 \end{aligned}$$

where the unknowns $\text {Q}_m \in \mathbb {R}^{P\times K}$ and $\text {Z}_m \in \mathbb {R}^{K\times P}$ are the mixing matrices, $\text {X}_{m,t} \in \mathbb {R}^{N\times K}$ and $\text {X}_{m,s} \in \mathbb {R}^{K\times V}$ contain the temporal and spatial features in the reduced dimension, respectively, and $\lambda _1/\lambda _2/\lambda _3$ are the sparsity regularization hyperparameters. For this article, both mixing matrices are assumed non-sparse, and hence their associated sparsity parameters $\lambda _1/\lambda _2$ can be ignored, and the simplified model is given as

5 $$\begin{aligned}{}&\min _{\text {C}_m, \text {Q}_m}\left\Vert \text {X}_{m,t} -\text {T}_p\text {C}_m\text {Q}_m\right\Vert _F^2, \quad \text {sub.to.} \ \ \left\Vert \text {T}_p\text {c}_{m,p}\right\Vert _2 =1, \quad \left\Vert \text {c}_{m,p}\right\Vert _0\le \zeta _1 \end{aligned}$$

6 $$\begin{aligned}{}&\min _{\text {S}_m, \text {Z}_m}\left\Vert \text {X}_{m,s} -\text {Z}_m\text {S}_m\right\Vert _F^2+\lambda _1\left\Vert \text {S}_m\right\Vert _1 \end{aligned}$$

The feature matrices are obtained using singular value decomposition (SVD), and the unknowns are solved using alternating least squares and soft thresholding via Neumann’s alternating projection lemma.

### Proposed algorithms

#### swbDL

In order to reinforce the fidelity of subject-wise recovered temporal dynamics, the autocorrelations of each dictionary atom at lag-1 are considered. Instead of directly incorporating the delayed time series^50^, the temporal correlation structure between the current and lagged dictionary is penalized so that for some sufficiently large $\alpha$, most of the entries that define the difference between the correlation structure $\alpha \left\Vert \text {D}_q\text {A}_m\text {A}_m^\top \text {D}_q^\top -\text {D}_0\text {D}_0^\top \right\Vert _F^2$ will shrink to zero. Thus, to update representation matrices $\text {A}_m$ and $\text {B}_m$, problem (3) can be modified as follows

7 $$\begin{aligned}{}&\min _{\text {A}_m, \text {B}_m}\left\Vert \text {Y}_m -\text {D}_q\text {A}_m\text {B}_m\text {X}_q\right\Vert _F^2+\alpha \left\Vert \text {D}_q\text {A}_m\text {A}_m^\top \text {D}_q^\top -\text {D}_0\text {D}_0^\top \right\Vert _F^2+\lambda _2\left\Vert \text {b}_m^k\text {X}_q\right\Vert _1, \nonumber \\&\quad \text {sub.to.} \ \ \left\Vert \text {D}_q\text {a}_k\right\Vert _2 =1 \end{aligned}$$

where $\text {D}_0$ is a time-delayed version of the original dictionary. To solve (7), a computationally efficient block update of representation matrices $\text {A}_m$ and $\text {B}_m$ is considered. Hence an alternating optimization approach is adopted where one of the two unknown variables is updated while the other is fixed. For this purpose, $\text {A}_m$ is fixed then the minimization objective reduces to

8 $$\begin{aligned}{}&\text {B}_m=\arg \min _{\text {B}_m}\left\Vert \text {Y}_m -\text {D}_q\text {A}_m\text {B}_m\text {X}_q\right\Vert _F^2+\lambda _2\left\Vert \text {b}_m^k\text {X}_q\right\Vert _1 \end{aligned}$$

The update for $\text {B}$ in Eq. (8) is obtained using soft thresholding and least squares as

9 $$\begin{aligned}{}&{\text {x}_m^k}= \textrm{sgn}\Big (\text {d}_{m,k}^\top \text {Y}_{m}\Big )\circ \Big (|\text {d}_{m,k}^\top \text {Y}_{m}|-\frac{\lambda _21}{2}\Big )_+ \nonumber \\&\text {B}_m = \text {X}_m\text {X}_q^\top \Big (\text {X}_q\text {X}_q^\top \Big )^{-1} \end{aligned}$$

Next, $\text {B}_m$ is fixed, $\text {D}_q\text {A}_m$ is replaced by $\text {D}_m$ to solve for it before obtaining an update for $\text {A}_m$, and the minimization function becomes

$$\begin{aligned}{}&\text {D}_m=\arg \min _{\text {D}_m}\left\Vert \text {Y}_m -\text {D}_m\text {B}_m\text {X}_q\right\Vert _F^2+\alpha \left\Vert \text {D}_m\text {D}_m^\top -\text {D}_0\text {D}_0^\top \right\Vert _F^2, \quad \text {sub.to.} \ \ \left\Vert \text {d}_m\right\Vert _2 =1 \end{aligned}$$

A relaxation variable $\text {U}$ is introduced in the above equation, and it is reformulated as

10 $$\begin{aligned}{}&\text {D}_m=\arg \min _{\text {D}_m}\frac{1}{2}\left\Vert \text {Y}_m -\text {D}_m\text {B}_m\text {X}_q\right\Vert _F^2+\frac{\alpha }{4}\left\Vert \text {UU}^\top -\text {D}_0\text {D}_0^\top \right\Vert _F^2, \quad \text {sub.to.} \ \ \left\Vert \text {D}_m-\text {U}\right\Vert _F^2 =0, \ \ \left\Vert \text {d}_m\right\Vert _2 =1 \end{aligned}$$

This can be solved using the ADMM algorithm that provides the closed-form solution for both $\text {D}_m$ and $\text {U}$, admits only one tuning parameter and converges for all of its positive values^60^. The augmented Lagrangian for Eq. (10) is given as

11 $$\begin{aligned}{}&\mathcalligra {L}_{\beta }(\text {D}_m,\text {U},\text {W})=\min _{\text {D}_m}\frac{1}{2}\left\Vert \text {Y}_m -\text {D}_m\text {B}_m\text {X}_q\right\Vert _F^2+\frac{\alpha }{4}\left\Vert \text {UU}^\top -\text {D}_0\text {D}_0^\top \right\Vert _F^2+\frac{\beta }{2}\left\Vert \text {D}_m-\text {U}\right\Vert _F^2+\textrm{tr}\Big [\text {W}^\top (\text {D}_m-\text {U})\Big ] \end{aligned}$$

where $\text {W}$ is the Lagrangian multiplier and $\beta$ is the tuning parameter. Due to normalization constraint on dictionary, all columns of $\text {D}_m$ and $\text {U}$ are normalized during each iteration. Initially setting $\text {U}$ and $\text {W}$ to zero, the solution to (11) is obtained by computing each of the following until convergence

12 $$\begin{aligned}{}&\text {D}_m=(\text {Y}_m\text {X}_m^\top +\beta \text {U}-\text {W})(\text {X}_m\text {X}_m^\top +\beta \text {I})^{-1}\nonumber \\&\text {U}=(\alpha \text {U}\text {U}^\top +\beta \text {I}-\alpha \text {D}_0\text {D}_0^\top )^{-1}(\beta \text {D}_m+\text {W})\nonumber \\&\text {W}=\text {W}+\beta (\text {D}_m-\text {U}) \end{aligned}$$

An update for $\text {A}_m$ is obtained during each iteration of the algorithm as

13 $$\begin{aligned}{}&\text {A}_m=(\text {D}_q^\top \text {D}_q)^{-1}\text {D}_q^\top \text {D}_m\nonumber \\&\text {a}_{m,k} = \text {a}_{m,k} /\left\Vert \text {D}_q\text {a}_{m,k}\right\Vert _2 \end{aligned}$$

The accompanying algorithm for swbDL is given in Table 1

**Table 1:**
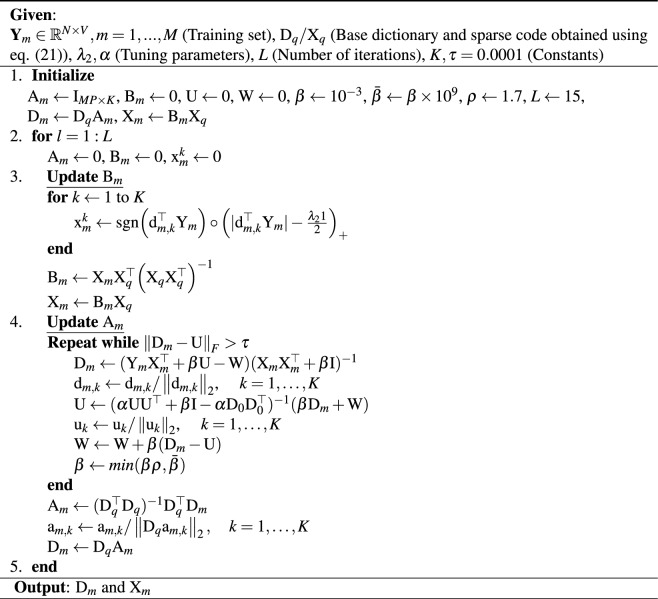
Algorithm for solving the minimization problem (7).

#### swsDL

In contrast, to block update, the sequential approach is presented in this section for a more precise update of the unknowns. To achieve this, instead of deploying the observed data matrix based decomposition, the rank-1 minimization problem based on the error matrix of all signals is considered where adaptive sparse penalty term is introduced for a fairer assignment of penalty to each entry in $\text {b}_m^k\text {x}_{q,j}$. Accordingly, to update representation matrices $\text {A}_m$ and $\text {B}_m$, problem (3) is reformulated as follows

14 $$\begin{aligned}{}&\{\text {a}_{m,k},\text {b}_m^k\}=\arg \min _{\text {a}_{m,k}, \text {b}_m^k}\left\Vert \text {E}_{m,k}-\text {D}_q\text {a}_{m,k}\text {b}_m^k\text {X}_q\right\Vert _{F}^2+\sum _{j=1}^{V}\lambda ^k_{2,j}|\text {b}_m^k\text {x}_{q,j}|, \nonumber \\&\quad \quad \quad \quad \quad \quad \text {sub.to.} \ \ \left\Vert \text {a}_{m,k}\right\Vert _0\le \zeta _2, \ \ \left\Vert \text {b}_m^k\right\Vert _0\le \zeta _3, \ \ \left\Vert \text {D}_q\text {a}_{m,k}\right\Vert _2 =1 \end{aligned}$$

where $\lambda ^k_{2,j}$ is a data-driven regularization parameter allocated to each entry of $\text {b}_m^k\text {X}_q$, and error matrix is

15 $$\begin{aligned} \text {E}_{m,k} = \text {Y}_m-\sum _{i=1,i\ne k}^{K}\text {d}_{m,i}\text {x}_m^i \end{aligned}$$

The $l_0$ constraint on representation matrices implements the regularization of dictionary atoms and sparse code through sparse basis expansion where bases have been constructed using components extracted from the ssBSS method. The update for $\text {a}_{m,k}$ and $\text {b}_m^k$ is obtained by solving the Lagrangian expression for (14) given as

16 $$\begin{aligned}{}&\mathcalligra {L}(\text {a}_{m,k},\text {b}_m^k)=\text {E}_{m,k}^\top \text {E}_{m,k}-2\text {E}_{m,k}^\top \text {D}_q\text {a}_{m,k}\text {b}_m^k\text {X}_q+\text {X}_q^\top {\text {b}_m^k}^\top \text {a}_{m,k}^\top \text {D}_q^\top \text {D}_q\text {a}_{m,k}\text {b}_m^k\text {X}_q+\sum _{j=1}^{V}\lambda ^k_{2,j}|\text {b}_m^k\text {x}_{q,j}| \end{aligned}$$

Solving this equation with respect to $\text {b}_m^k$ we obtain

$$\begin{aligned}{}&-2\text {a}_{m,k}^\top \text {D}_q^\top \text {E}_{m,k}\text {X}_q^\top +2\text {a}_{m,k}^\top \text {D}_q^\top \text {D}_q\text {a}_{m,k}\text {b}_m^k\text {X}_q\text {X}_q^\top +\sum _{j=1}^{V}\lambda ^k_{2,j}\frac{d|\text {b}_m^k\text {x}_{q,j}|}{db_m^k}=0 \end{aligned}$$

Because $\text {d}_{m,k}=\text {D}_q\text {a}_{m,k}$ due to the definition, $\text {d}_{m,k}^\top \text {d}_{m,k}=1$ due to normalization constraint on dictionary columns, and $\text {x}_{q,j}$ can be extracted out of the third term as its a constant, then the above equation further unfolds as

$$\begin{aligned}{}&\text {b}_m^k\text {X}_q\text {X}_q^\top =\text {d}_{m,k}^\top \text {E}_{m,k}\text {X}_q^\top -\frac{1}{2}\sum _{j=1}^{V}\lambda ^k_{2,j}\frac{d|\text {b}_m^k|}{{db_m^k}} \sum _{j=1}^{V}|\text {x}_{q,j}| \end{aligned}$$

Considering $$\text {X}_q^\top =\sum _{j=1}^{V}|\text {x}_{q,j}|$$ then

$$\begin{aligned}{}&\text {b}_m^k\text {X}_q\text {X}_q^\top =\text {d}_{m,k}^\top \text {E}_{m,k}\text {X}_q^\top -\frac{1}{2}\sum _{j=1}^{V}\lambda ^k_{2,j}\frac{d|\text {b}_m^k|}{{db_m^k}} \text {X}_q^\top \\&\text {b}_m^k\text {X}_q\text {X}_q^\top =\Bigg (\text {d}_{m,k}^\top \text {E}_{m,k}-\frac{1}{2}\sum _{j=1}^{V}\lambda ^k_{2,j}\frac{d|\text {b}_m^k|}{{db_m^k}}\Bigg ) \text {X}_q^\top \\&\text {b}_m^k=\Bigg (\text {d}_{m,k}^\top \text {E}_{m,k}-\frac{1}{2}\sum _{j=1}^{V}\lambda ^k_{2,j}\frac{d|\text {b}_m^k|}{{db_m^k}}\Bigg ) \text {X}_q^\top \Big (\text {X}_q\text {X}_q^\top \Big )^{-1} \end{aligned}$$

This can be further simplified using the soft thresholding approach^59^ as

$$\begin{aligned}{}&\text {b}_m^k=\textrm{sgn}\Big (\text {d}_{m,k}^\top \text {E}_{m,k}\Big )\circ \Big (|\text {d}_{m,k}^\top \text {E}_{m,k}|-\frac{\lambda _{2}1}{2|\text {d}_{m,k}^\top \text {E}_{m,k}|}\Big )_+\text {X}_q^\top \Big (\text {X}_q\text {X}_q^\top \Big )^{-1} \end{aligned}$$

which can be rewritten in simplified form as

17 $$\begin{aligned}{}&\text {b}_m^k=\text {x}_m^k\text {X}_q^\top \Big (\text {X}_q\text {X}_q^\top \Big )^{-1} \end{aligned}$$

where $\text {x}_m^k=\textrm{sgn}\Big (\xi ^k\Big )\circ \Big (|\xi ^k|-\frac{\lambda _{2}1}{2|\xi ^k|}\Big )_+$, $\xi ^k=\text {d}_{m,k}^\top \text {E}_{m,k}$, and $\lambda _2$ is a scalar tuning parameter. By taking into account the $l_0$ norm imposed on $\text {b}_m^k$ the Eq. (17) is reformulated as a constrained problem

18 $$\begin{aligned}{}&{\text {b}_m^k}=\arg \min _{\text {b}_m^k}\left\Vert \text {x}_m^k-\text {b}_m^k\text {X}_q\right\Vert _2^2 \quad \text {sub.to.} \ \left\Vert \text {b}_m^k\right\Vert _0\le \zeta _3 \end{aligned}$$

This can be solved as $\text {b}^k_{m,\vartheta }=\text {x}_m^k{\text {X}^{\vartheta }_q}^\top \Big (\text {X}^{\vartheta }_q{\text {X}^{\vartheta }_q}^\top \Big )^{-1}$, where the indices set $\vartheta$ can be found using thresholded correlation values whose related algorithm is described in^50,61,62^. Solving Eq. (16) with respect to $\text {a}_{m,k}$, following solution is obtained

19 $$\begin{aligned}{}&-2\text {D}_q^\top \text {E}_{m,k}\text {X}_q^\top \text {b}_m^{k^\top }+2\text {D}_q^\top \text {D}_q\text {a}_{m,k}\text {b}_m^k\text {X}_q\text {X}_q^\top \text {b}_m^{k^\top }=0\nonumber \\&\implies {\text {a}_{m,k}=(\text {D}_q^\top \text {D}_q)^{-1}\text {D}_q^\top \text {d*}_{m,k}} \end{aligned}$$

where $\text {d*}_{m,k}=\text {d}_{m,k}+1/\text {x}_m^k\text {x}_m^{k^\top }\Big (\text {E}_{m,k}\text {x}_m^{k^\top }\Big )$, $\text {d}_{m,k}=\text {D}_q\text {a}_{m,k}$, and $\text {x}_m^k=\text {b}_m^k\text {X}_q$. By considering the $l_0$ norm on $\text {a}_{m,k}$ the Eq. (19) as a constrained problem is given as

20 $$\begin{aligned}{}&{\text {a}_{m,k}}=\arg \min _{\text {a}_{m,k}}\left\Vert \text {d}_{m,k}-\text {D}_q\text 
{a}_{m,k}\right\Vert _2^2 \quad 
\text {sub.to.} \ \left\Vert \text {a}_{m,k}\right\Vert _0\le \zeta _2 \end{aligned}$$

This implies $\text {a}^{\vartheta }_{m,k}=(\text {D}_{q,\vartheta }^\top \text {D}_{q,\vartheta })^{-1}\text {D}_{q,\vartheta }^\top \text {d}_{m,k,\varepsilon }$, where $\text {d}_{m,k,\varepsilon }=\text {d}_{m,k}+1/\text {x}_{m,\varepsilon }^k\text {x}_{m,\varepsilon }^{k^\top }\Big (\text {E}_{m,k,\varepsilon }\text {x}_{m,\varepsilon }^{k^\top }\Big )$ admits only the non-zero entries of the coefficient row through indices set $\varepsilon$^50^ leading to a reduced error matrix and reduced computational cost. The swsDL algorithm is described in Table 2.

**Table 2:**
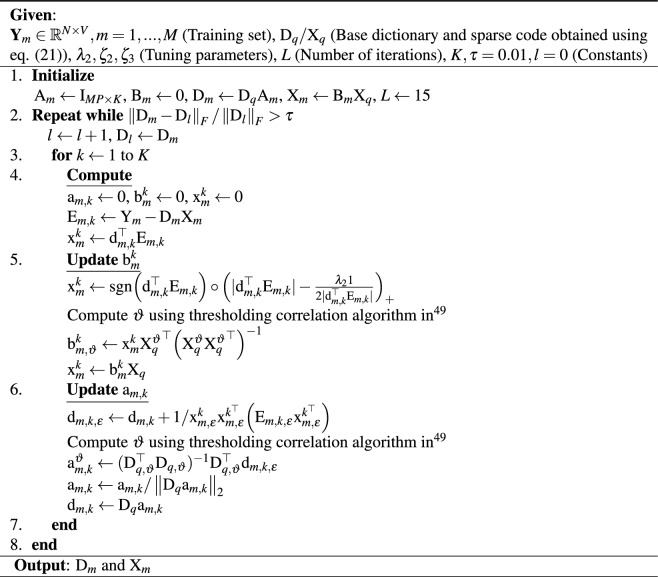
Algorithm for solving the minimization problem (14).

### Proposed framework

Since the proposed model is applied to multiple subjects, group-level analysis was also implemented in addition to the subject-wise inferences. In this context, (i) modeled HRF (MHRs) were produced by using the convolution operation between the task stimuli and canonical HRF from the statistical parametric mapping (SPM) toolbox^63^, and (ii) resting-state network templates (RSNs) (R1-R10) were obtained from Smith^64^. While both MHRs and RSNs were used to accomplish group analysis of task-related data, only RSNs were used for group analysis of resting-state data. The following steps were involved in achieving both subject-wise and group-level analysis

1. *Preparing the preliminary bases:* Source components $\text {T}_m$ and $\text {S}_m$ obtained using the fast ssBSS method were concatenated along the spatial (horizontal) and temporal (vertical) dimension, respectively, to construct base dictionary $\text {D}_q$ and base sparse code $\text {X}_q$ as
  21 $$\begin{aligned}{}&\text {D}_q = [\text {T}_1, \text {T}_2, \ldots , \text {T}_M]\nonumber \\&\text {X}_q = [\text {S}_1^\top , \text {S}_2^\top , \ldots , \text {S}_M^\top ]^\top \end{aligned}$$
2. *Performing subject-wise analysis:* Subject-wise $\text {D}_m/\text {X}_m$ were trained using either swbDL or swsDL algorithm as described in the previous section.
3. *Performing group-level analysis:* For this, the following scenarios were considered
  - For task-related stimuli, the atoms in all subject-wise dictionaries that were most correlated with MHRs were assembled along with their corresponding sparse codes in matrices $\text {D}_r/\text {X}_r$ to obtain group-level dynamics through rank-1 decomposition via SVD as follows
    22 $$\begin{aligned}{}&\text {D}_r = [\text {d}_{1,j_1(r)}, \text {d}_{2,j_2(r)},\ldots ,\text {d}_{M,j_M(r)}]\nonumber \\&\text {X}_r = \Big [\text {x}_1^{j_1(r)^\top }, \text {x}_2^{j_2(r)^\top },\ldots ,\text {x}_M^{j_M(r)^\top }\Big ]^\top \nonumber \\&\frac{1}{M}[\text {D}_{r}\text {X}_r]=\omega _r\delta _r\gamma _r^\top \nonumber \\&\text {d}_{g,r} = \omega _r \nonumber \\&\text {x}_{g}^r = \delta _r\gamma _r^\top \end{aligned}$$
    where $r =\{1,\ldots ,R\}$, *R* is the number of MHRs, $j_m(r)$ represents the indices of the most correlated atom in *m*-th dictionary with *r*-th MHR, $m=\{1,\ldots ,M\}$, *M* is the number of subjects, and $\text {D}_g$ is the group-level dictionary.
  - For resting-state networks, the subject-wise sparse code rows from all subjects that were most correlated with RSNs were assembled as
    23 $$\begin{aligned}{}&\Pi _r=\Big [|\text {x}_1^{j_1(r)^\top }|, |\text {x}_2^{j_1(r)^\top }|, \ldots , |\text {x}_M^{j_M(r)^\top }|\Big ]^\top \nonumber \\&\text {x}_g^r=\frac{1}{M}\sum _{m=1}^{M} \pi _r^m \end{aligned}$$
    where $j_m(r)$ represents the indices of the most correlated coefficient row in *m*-th sparse code matrix with *r*-th RSN, and $\text {X}_g$ is the group-level sparse code.

**Figure 2:**
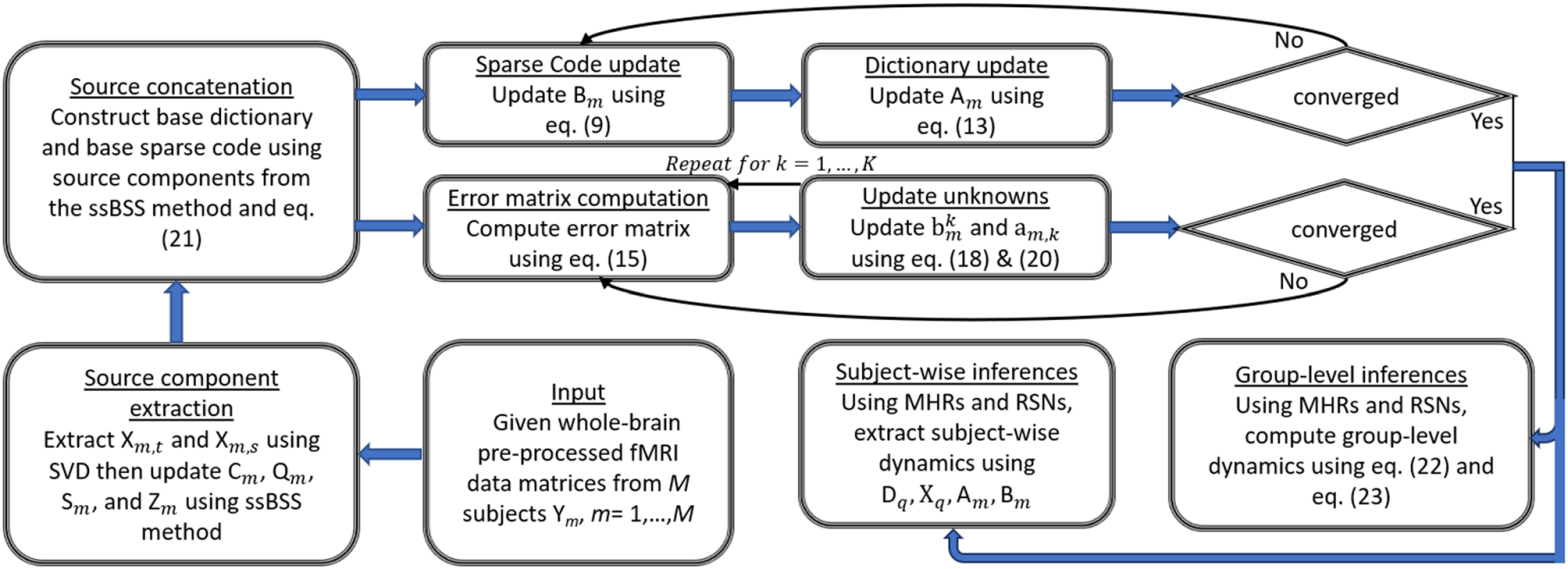
A block diagram of the proposed framework where the ssBSS method extracts the base components and the rest of the blocks attempt to recover subject-wise and group-level TCs and SMs either block-wise or sequentially.

The proposed framework in form of a block diagram is given in Fig. 2.

### Ethical approval

This study’s block-design and resting-state fMRI datasets are open-access and shared on the human connectome website https://www.humanconnectome.org/study/hcp-young-adult. The condition for using these datasets is properly acknowledging the funding source and citing relevant publications, which we have in the acknowledgment and experiment sections. Therefore, we do not need any approval from the ethics committee of respective institutes.

## Experiments

This section evaluates the proposed algorithms to determine their capability compared to the existing state-of-the-art data-driven algorithms. For this purpose, data analysis was conducted using three different fMRI datasets, one synthetic and two experimental. The participating algorithms are cgICA, sgICA, CODL, ACSD, ssBSS, swbDL, and swsDL. The main reason for not incorporating sgBACES and ShSSDL algorithms in the comparison study is their inability to extract subject-wise dynamics. The Simtb toolbox^65^ was utilized to generate the synthetic fMRI dataset of four subjects. The Human Connectome Project (HCP)^66,67^ was availed to acquire eight subject’s block design fMRI dataset from its quarter 3 release, and eight subject’s resting-state fMRI dataset from its S500 and S900 release. These datasets allowed us to assess the performance of all participating algorithms in terms of their potential to retrieve the ground truth.

### Synthetic dataset

**Figure 3:**
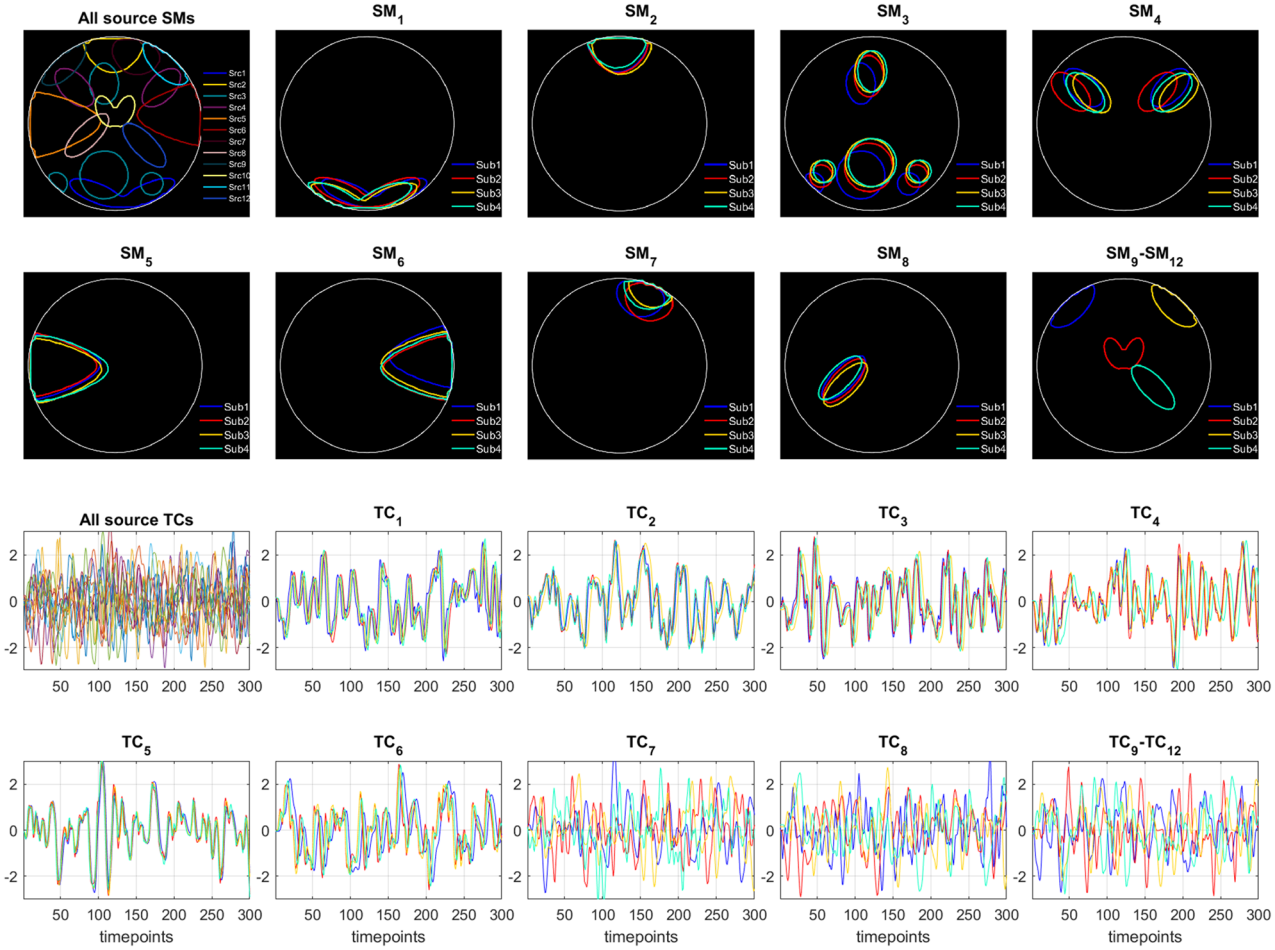
The top two rows show all spatial sources with respect to the first subject, the location and shape of each of the twelve spatial sources, and their variability across subjects. In contrast, the bottom two rows show the respective temporal sources where $\textrm{TC}_7$ and $\textrm{TC}_8$ have four unique patterns.

In this section, a realistic fMRI dataset of four subjects was generated using the Simtb toolbox. Eighteen distinct temporal sources, each consisting of 300 timepoints with a repetition time (TR = 1 $\textrm{sec}$) and twelve distinct spatial sources, each consisting of size $50\times 50$ voxels, were used to obtain these four datasets. The source IDs for the spatial components were set to $\{3, 6, 8, 10, 22, 23, 26, 30, 4, 12, 5, 29\}$. Overall, nine spatiotemporal sources out of all source signals were used to generate each subject’s fMRI data. With some variability across subjects, the first six temporal and six respective spatial sources were present in all subjects; the following two spatial sources were also common, but their temporal patterns were unique to each subject, and the last source’s both spatial and temporal features were unique to each subject as shown in Fig. 3.

**Figure 4:**
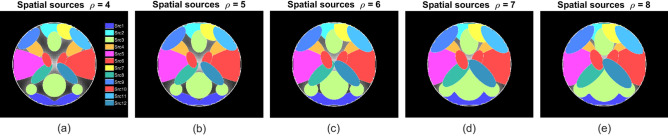
Using five different values of the spread parameter $\rho$, moderate to substantial spatial overlaps were created by controlling the size of twelve different activation blobs.

For common temporal sources, the variability across subjects was introduced by varying the HRF parameters, such as delay/dispersion of response/undershoot. Similarly, the intersubject variability for the common spatial maps was established by using parameters of the Gaussian distribution (mean ($\mu$) and standard deviation (std) ($\sigma$)) that allowed controlling the location, orientation, and spread of the activations. This was realized by random translation in x and y direction ($\mu =0, \sigma =1.5$), random rotation ($\mu =0, \sigma =0.9$), and random scaling ($\mu =\rho , \sigma =0.05$) as shown in Fig. 3. Here $\rho$, the Gaussian distribution’s mean, is considered the spread parameter using which the spatial extent of the activations was controlled to create five unique cases of spatial overlaps. The corresponding spatial maps with moderate to substantial dependence are shown in Fig. 4a–e.

The first subject’s common and unique spatiotemporal sources and three other unique spatiotemporal sources from the remaining three subjects are assembled and treated as the ground truth TCs and SMs for group-level analysis as shown in Fig. 3 under the heading all source SMs/all source TCs. The other subfigures show the spatial and temporal sources that are used to generate all four datasets where each of the main temporal sources $\textrm{TC}_7$ and $\textrm{TC}_8$ consists of four unique temporal patterns. Using a linear mixture model, these sources were utilized to generate each subject’s dataset as $\text {Y}=\sum _{i=1}^{8}(\text {tc}_i+\psi _i)(\text {sm}^i+\phi ^i)$, where the noise generating matrices $\Psi \in \mathbb {R}^{300\times 9}$ and $\Phi \in \mathbb {R}^{9\times 2500}$ were produced using Gaussian distribution $\sim \mathcal {N}(0,\,\sigma ^2=n_t)$ and $\sim \mathcal {N}(0,\,0.01)$, respectively, where $n_t$ represents the variance of the temporal noise. Depending on the value of $\rho$, $n_t$, and trial number, the datasets $\text {Y}_{m=1}^M$ (where $M=4$) were then produced and employed by all algorithms for source retrieval.

### Synthetic dataset dictionary learning

The parameter values were kept consistent across all algorithms wherever feasible to produce a fair comparison. In contrast to experimental fMRI data, the ground truth about the number of source signals is known; therefore, the same number of components as the number of generating sources were trained for the simulated dataset. Since the cgICA/sgICA/CODL were applied to the grouped data, the total number of components to be extracted was set to 12. In comparison, it was set to 9 for ACSD/ssBSS/swbDL/swsDL, which were applied subject-wise. In contrast to CODL, which iterated for 30 iterations, all other dictionary learning algorithms, including ssBSS, were run for 15 iterations. After evaluating different strategies, the optimal dictionary initialization for each algorithm was supplied. Concatenated data, random numbers drawn from the standard normal distribution, and DCT bases were employed for CODL, ssBSS, and ACSD/swbDL/swsDL, respectively.

The tuning parameters were handled by experimenting with their various combinations. Those values were considered that produce the best results in terms of similarity between the recovered sources and their respective ground truth. Twelve components were kept after each of the two PCA reductions in the case of cgICA/sgICA. The best sparsity and smoothing parameter for sgICA was 3 and 50000, respectively. For a fair comparison with other dictionary learning methods, CODL’s batch size was adjusted to $b=2500$, and its temporal reduction was avoided, whereas its sparsity parameter was set to 1.5. For ACSD, the best sparsity parameter was found to be 12. For ssBSS, the tuning parameters were set as $\lambda _1=6$ and $\zeta _1=30$, $K_p=150$, nine components were obtained from PCA, and nine were retained for iterative routine. For swbDL, the tuning parameters were set as $\lambda _2=8$ and $\alpha =1$. For swsDL, the best sparsity parameters were found to be $\zeta _2=\zeta _3=24$ and $\lambda _2=16$.

### Synthetic dataset results

The multi-subject dataset generation and the learning process were repeated several times for different noise realizations to demonstrate the robustness and consistency of the proposed algorithms. To achieve this, the experiment, which included both data generation and learning process, was repeated for (i) two different variance (square of the standard deviation) values of the temporal noise set as $n_t=\{0.3, 0.9\}$, (ii) five values of $\rho$ that were varied from 4 to 8 to gradually increase activation overlaps as shown in Fig. 4a–e, and (iii) 150 different trials.

**Figure 5:**
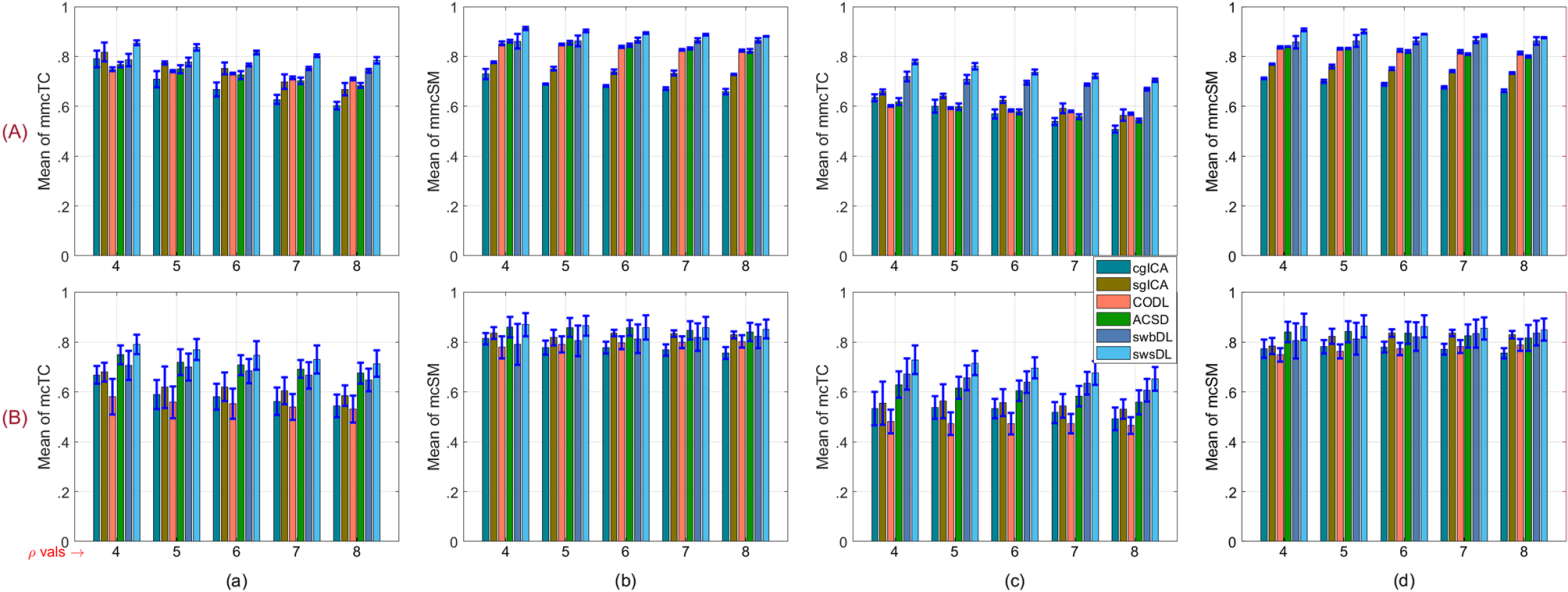
For noise variance 0.3/0.9, (**A**) subject-wise analysis’s mean values of (a)/(c) mmcTC and (b)/(d) mmcSM computed over 9 components, 4 subjects, and 150 realizations, and (**B**) group-level analysis’s mean values of (a)/(c) mcTC and (b)/(d) mcSM computed over 9 components and 150 realizations. The deviation from the mean values has been plotted as error bars.

**Figure 6:**
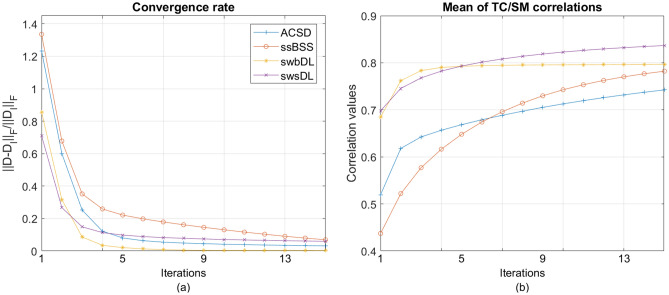
Over all trials, spatial overlap cases, noise realizations, and subjects, the mean of the (**a**) convergence rate and (**b**) correlation values between the ground truth and retrieved sources for subject-wise dictionary learning shown as a function of algorithm iterations.

**Figure 7:**
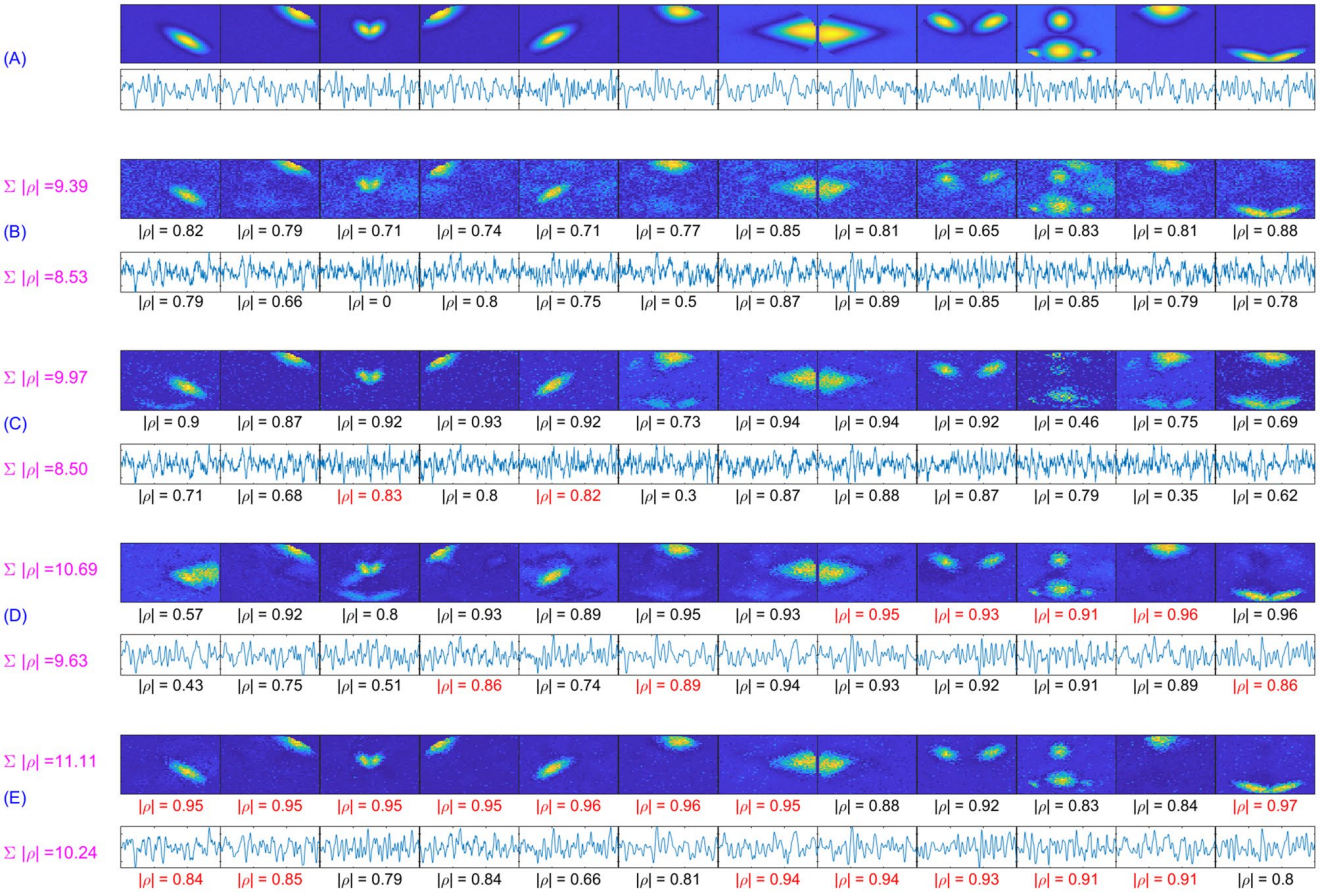
(**A**) Ground truth TCs/SMs, and the recovered TCs/SMs by (**B**) sgICA, (**C**) ACSD, (**D**) swbDL, and (**E**) swsDL, along with the absolute temporal and spatial correlation values ($\gamma$) for each source, and the sum of these correlation values shown on the left. The highest correlation value for each source is shown in a different colour.

Moreover, the source recovery was performed in regards to both subject-wise and group-wise analysis. Underlying source TCs/SMs were obtained by keeping the indices with the highest correlation values after correlating every algorithm’s trained dictionary atoms/sparse code rows with the ground truth TCs/SMs. These correlation values were computed with respect to ground truth SMs and are retained as cTC/sSM. For each of the five spatial overlap scenarios and two noise realizations, the mean of the cTC/cSM values over all nine spatiotemporal sources are saved as mcTC/mcSM, their mean mmcTC/mmcSM over 4 subjects, and the mean of mmcTC/mmcSM over 150 trials are plotted in Fig. 5A for subject-wise analysis, and in Fig. 5B for group-wise analysis (where the mean of correlation values over the subjects has been excluded). The convergence rate and the progression of correlation values for the proposed algorithms as functions of algorithm iterations are shown in Fig. 6. The component-wise visual comparison among participating algorithms for source recovery is provided in Fig. 7.

From Fig. 5, one can conclude that the swsDL algorithm consistently outperformed all other algorithms for all source recovery scenarios, including spatial/temporal feature, subject-wise/group-level analysis, and spatial overlap cases. It attained the highest recovery performance for low noise levels and spatial dependence. Although this performance dropped as noise intensity and spatial overlaps increased, it remained superior to all other competing algorithms. Its block variant swbDL emerged as a runner-up for the subject-wise analysis, whereas ACSD seems to have replaced its runner-up position for the group-level analysis. Moreover, sgICA has outperformed cgICA by exhibiting lower standard deviation and better recovery precision. The sgICA, for high spatial overlap and noise variance, even surpassed the ACSD algorithm for group-level analysis. It is also noticeable that both proposed algorithms performed relatively superior for subject-wise analysis.

It can be deduced from Fig. 6a that the swbDL algorithm, compared to ssBSS, ACSD and swsDL, converged faster and only needed a few iterations to produce the desired results. This trend also manifests in Fig. 6b, where the correlation strength nearly stopped accumulating for swbDL after the fifth iteration. In contrast, ssBSS, ACSD and swsDL algorithms converged slowly and showed source recovery improvement as the number of iterations increased.

In order to produce component-wise visual comparison, the experiment was repeated for subject-wise analysis using parameter settings as $n_t=0.3$ and $\rho =5$. For swbDL, the tuning parameter value was changed to $\alpha =0.75$. Due to a lack of space and inferior results from CODL and cgICA, they have been dropped for this particular case. The results were extracted for all four subjects; however, the first nine components were selected from subject 1, and components $\{10,11,12\}$ belonged to subject $\{2,3,4\}$ ninth component. The results of this experiment are shown in Fig. 7. It can be depicted from this figure that swsDL defeated all other algorithms in source recovery strength for both spatial and temporal features while swbDL was the second best. The correlation values are written at the bottom of each source, with the best values highlighted in red.

### Experimental fMRI dataset

#### Experimental fMRI dataset preprocessing

The HCP had already preprocessed the resting-state datasets using their preprocessing tools; therefore, they were excluded from our preprocessing routine. On the other hand, block design datasets were preprocessed using the SPM-12 toolbox^63^. The steps involved in the preprocessing of this dataset, such as realignment, normalization, spatial smoothing, and masking, are described in depth in^50,68,69^. Firstly, functional images were realigned to the first image to remove motion artifacts. Secondly, all images were spatially normalized to a Tailarach template, resampled to $2 \times 2 \times 2$ $\textrm{mm}^3$ voxels, and spatially smoothed using a $6\times 6 \times 6$ $\textrm{mm}^3$ full-width at half-maximum (FWHM) Gaussian kernel. Thirdly, the masking step attempts to remove any data outside the scalp. Next, for each subject, the four-dimensional dataset was reshaped and saved as a 2-dimensional matrix called $\text {Y}_m$ to be considered a whole brain dataset, where $m=\{1,\ldots ,8\}$. This resulted in the size of each subject’s $\text {Y}$ matrix being $279 \times 236115$ for the block design dataset and $400 \times 230367$ for the resting-state dataset. Temporal filtering was performed on both types of datasets as the next step. This consisted of DCT based high-pass filter to remove low-frequency trends and FWHM based low-pass filter to eliminate high-frequency physiological noise. The cutoff for a DCT filter was set to 1/150 $\text {Hz}$ for block design and 1/128 $\text {Hz}$ for resting-state datasets, and a cutoff for FWHM was set to $1\ \textrm{sec}$ for both block design and resting-state datasets. After executing the aforementioned steps, all columns of $\text {Y}$ were normalized to have zero mean and unit variance.

#### Block design dataset

In this section, we employed the motor task 3T MRI raw block design dataset obtained from the quarter 3 release of the HCP^66,67^. An experiment was conducted for 204 $\textrm{secs}$ to acquire this dataset to map the brain’s motor cortex. During the experiment, the subjects were instructed to tap their right or left fingers, pinch their right or left toes, or move their tongues in response to visual stimuli. Following a three-second visual cue, subjects underwent a specific movement task lasting 12 seconds. Ten movement tasks were considered consisting of two tongue motions, left/right finger, and left/right toe movements. As a result, there were a total of 13 blocks, including three fixation blocks of $15 \ \textrm{secs}$. Six modeled HRFs (MHRs) were created utilizing the canonical HRF and task stimuli associated with five different movement types: left toe (LT), left finger (LF), right toe (RT), right finger (RF), tongue (T), and visual type cue (VC) to acquire ground truth TCs. Each subject had their fMRI scans taken using a Siemens 3 Tesla (3T) scanner. The acquisition’s specifications were echo time (TE) = $33.1 \ \textrm{ms}$, TR = $0.72 \ \textrm{secs}$, field of view (FOV) = $208 \times 180 \ \textrm{mm}$, flip angle (FA) = $52^o$, matrix size = $104 \times 90$, slice thickness = $2 \ \textrm{mm}$ with 72 contiguous slices, and $2 \ \textrm{mm}$ isotropic voxels, BW = $2290 \ \mathrm {Hz/Px}$, echo spacing = $0.58 \ \textrm{ms}$, and 284 EPI volumes were collected where first 5 were considered dummy and discarded. The block design dataset of eight subjects aged between 22 and 35 years was used in our analysis.

#### Block design dataset dictionary/component learning

For dictionary initialization, concatenated data, random numbers, and DCT bases were employed for CODL, ssBSS, and ACSD/swbDL/swsDL, respectively. The total number of iterations for all dictionary learning algorithms was set to 15 except for CODL and ssBSS, for which this number was set to 30. While performing dimensionality reduction, 100 components were kept from PCA, and 60 were retained when PCA was applied for the second time, and these many were extracted using cgICA and sgICA. These numbers and other parameters in this section were selected after trying their different combinations and considering that the selected ones must produce the best results in terms of correlation strength between the retrieved source and the ground truth. For sgICA, the sparsity parameter was set to 5 while the smoothing parameter was 50000. The total number of dictionary atoms to be trained using CODL was set to 70 with the sparsity parameter set to 6 with batch size equal to the data dimension. Using ACSD, 40 dictionary atoms were trained for each subject with a sparsity parameter set to 60. For ssBSS, 60 components were retained from PCA, and 40 were trained; its other parameters were set as $\lambda _1=16$, $\zeta _1=50$, and $K_p=60$. For both swbDL and swsDL, 40 atoms were trained, tuning parameters were set as $\lambda _2=12$ and $\alpha =3000$ for swbDL, and tuning parameters were set to $\zeta _2=\zeta _3=48$ and $\lambda _2=25$ for swsDL.

#### Block design dataset results

In this section, the absence of activation maps for task-related components encouraged us to choose temporal analysis using six constructed MHRs. Similarly, the absence of temporal profiles for resting state networks motivated us to choose some of Smith’s templates from R1-R10. The analysis was based on two strategies, i) subject-wise and ii) group-level. For subject-wise analysis, the TCs/SMs obtained by ACSD, ssBSS, swbDL, and swsDL for each subject were considered, whereas individual TCs/SMs for cgICA, sgICA, and CODL was obtained by back reconstruction. In contrast, for the group-level analysis, the group-level TCs/SMs obtained by all competing algorithms were used as a reference for further evaluation. Eventually, these TCs were correlated with the MHRs and SMs with RSNs, and the highest correlation values and the respective atoms/sparse codes were saved. Group-level correlation values are specified in Table 3, and the average correlation values over all subjects are mentioned in Table 4. The highest values in these tables have been highlighted in bold.

**Table 3:** For the block design dataset, correlation values of the most correlated group-level dictionary atom with six MHRs and most correlated common spatial maps with RSN templates obtained using seven competing algorithms, including the proposed.

Algos	VC	LT	LF	RT	RF	T	R1	R2	R3	R4	R9	R10	Mean
cgICA	0.838	0.909	0.864	0.758	0.838	0.889	0.689	0.596	0.393	0.412	0.392	0.402	0.665
sgICA	0.846	0.911	0.863	0.775	0.829	0.882	0.684	0.588	0.412	0.522	0.425	**0.524**	0.688
CODL	0.913	0.887	0.837	0.754	0.816	0.876	**0.709**	0.602	0.369	0.488	0.372	0.374	0.666
ACSD	0.884	0.750	0.832	0.601	0.831	0.843	0.632	0.726	0.442	**0.556**	0.434	0.482	0.668
ssBSS	0.902	0.840	0.855	0.731	0.844	0.857	0.471	0.737	0.462	0.481	0.403	0.474	0.671
swbDL	0.914	**0.939**	**0.872**	0.790	0.853	0.875	0.543	0.742	0.458	0.481	0.437	0.474	0.698
swsDL	**0.940**	0.920	0.867	**0.833**	**0.859**	**0.895**	0.570	**0.753**	**0.465**	0.519	**0.441**	0.441	**0.709**

**Table 4:** For six different block design tasks, correlation values of the most correlated back reconstructed component/atom with MHRs using cgICA/sgICA/CODL and averaged correlation values over the most correlated subject-level dictionary atom with MHRs obtained using ACSD/ssBSS/swbDL/swsDL.

Algos	VC	LT	LF	RT	RF	T	Mean
cgICA	0.628	0.715	0.741	0.610	0.674	**0.788**	0.693
sgICA	0.626	0.711	0.731	0.635	0.664	0.777	0.691
CODL	0.731	0.743	0.685	0.630	0.667	0.757	0.702
ACSD	0.680	0.574	0.607	0.455	0.611	0.729	0.610
ssBSS	0.712	0.680	0.688	0.483	0.642	0.671	0.646
swbDL	0.757	0.815	0.718	0.657	0.698	0.695	0.723
swsDL	**0.831**	**0.819**	**0.752**	**0.711**	**0.749**	0.785	**0.775**

#### Resting state dataset

The resting-state dataset of all participating subjects was acquired from the first set of 3T MRI preprocessed S500 and S900 release of the HCP^66,67^. The resting-state dataset was obtained using acquisition parameters identical to the block design dataset. During the experiment, subjects were instructed to maintain fixation on a bright crosshair displayed in a darkened room, and 1200 scans were recorded twice in a single session for two different phase encoding directions. The second run, which featured left-to-right phase encoding, was considered for our study. Only 400 scans were kept for analysis, while the first 20 and the last 780 scans were discarded. The preprocessed resting-state data also went through spatial smoothing using $6 \times 6 \times 6 \ \mathrm {mm^3}$ FWHM Gaussian kernel, followed by temporal smoothing using DCT and temporal FWHM filter. The resting-sate dataset of eight subjects aged between 26 and 35 years was used in our analysis.

#### Resting state dataset dictionary/component learning

Similar to synthetic and block design datasets, concatenated data, random numbers, and DCT bases were deployed for CODL, ssBSS, and ACSD/swbDL/swsDL, respectively, while initializing their respective dictionaries. Both CODL and ssBSS were iterated for 30 iterations due to their slow convergence, whereas ACSD/swbDL/swsDL were run for 15. For cgICA and sgICA, 100 components were retained using PCA, followed by keeping/extracting 50 components for the second PCA and ICA/sICA algorithm. Similar to the last two datasets, this section’s parameter selection also depended on the correlation strength between the recovered sources and the ground truth. The sparsity parameter was set to 5 and the smoothing parameter to 30000 for sgICA. The number of dictionary atoms for CODL was set to 70, the sparsity parameter to 6, and the batch size equal to the data dimension. For ACSD, 40 dictionary atoms were trained with sparsity parameters set to 30 for each subject. For ssBSS, 60 components were preserved from PCA, and 40 were trained; its tuning parameters were set as $\lambda _1=10$, $\zeta _1=90$, and $K_p=150$. For both swbDL and swsDL, 40 atoms were trained, tuning parameters were set as $\lambda _2=10$ and $\alpha =500$ for swbDL, and tuning parameters were set to $\zeta _2=\zeta _3=80$ and $\lambda _2=50$ for swsDL.

#### Resting state dataset results

Due to the absence of task-related components, our analysis in this section was based solely on Smith’s resting state templates^64^. Similar to the previous two datasets, this study was also conducted for two different scenarios (i) subject-wise and (ii) group-wise. For subject-wise analysis, the SMs obtained using subject-wise ACSD, ssBSS, swbDL, and swsDL were taken into account, and for cgICA, sgICA, and CODL group-level SMs were considered to back reconstruct individual SMs. In contrast, for the group-level analysis, the shared SMs obtained by all competing algorithms were used as a reference for further evaluation. Eventually, these SMs were correlated with the RSN templates, the highest correlation values, and the respective atoms/sparse codes were saved. Group-level correlation values, along with their mean, are specified in Table 5 where the highest values have been highlighted in bold.

**Table 5:** For resting-sate data, correlation values of the most correlated common spatial maps with RSN templates obtained using seven competing algorithms, including the proposed.

Algos	R1	R2	R3	R4	R5	R6	R7	R8	R9	R10	Mean
cgICA	0.754	0.657	0.604	0.517	0.313	0.381	0.353	0.460	0.410	0.528	0.497
sgICA	0.758	0.656	0.673	0.474	0.224	0.402	0.340	0.466	0.530	0.579	0.510
CODL	**0.837**	0.694	0.527	0.652	**0.456**	0.350	0.458	0.475	0.388	0.458	0.529
ACSD	0.704	0.687	0.571	0.675	0.365	0.436	0.463	0.446	0.530	0.579	0.546
ssBSS	0.694	0.693	0.649	0.680	0.308	**0.462**	0.506	0.467	0.542	0.610	0.561
swbDL	0.725	**0.741**	0.635	0.697	0.380	0.445	0.502	0.428	0.587	**0.634**	0.577
swsDL	0.758	0.739	**0.685**	**0.712**	0.393	0.421	**0.543**	**0.492**	**0.591**	0.608	**0.594**

**Figure 8:**
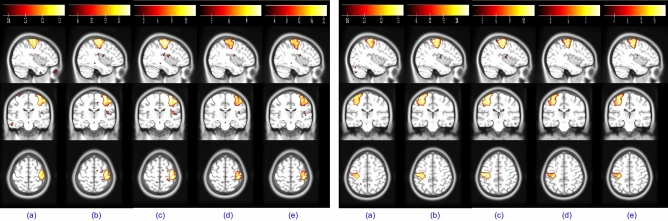
The thresholded fourth subject’s activation maps at a random field correction $p<0.001$ extracted for the left and right finger movement tasks of the block design dataset using (**a**) sgICA, (**b**) CODL, (**c**) ACSD, (**d**) swbDL, and (**e**) swsDL, respectively. Table 4 provides the related averaged correlation values.

### Discussion

**Figure 9:**
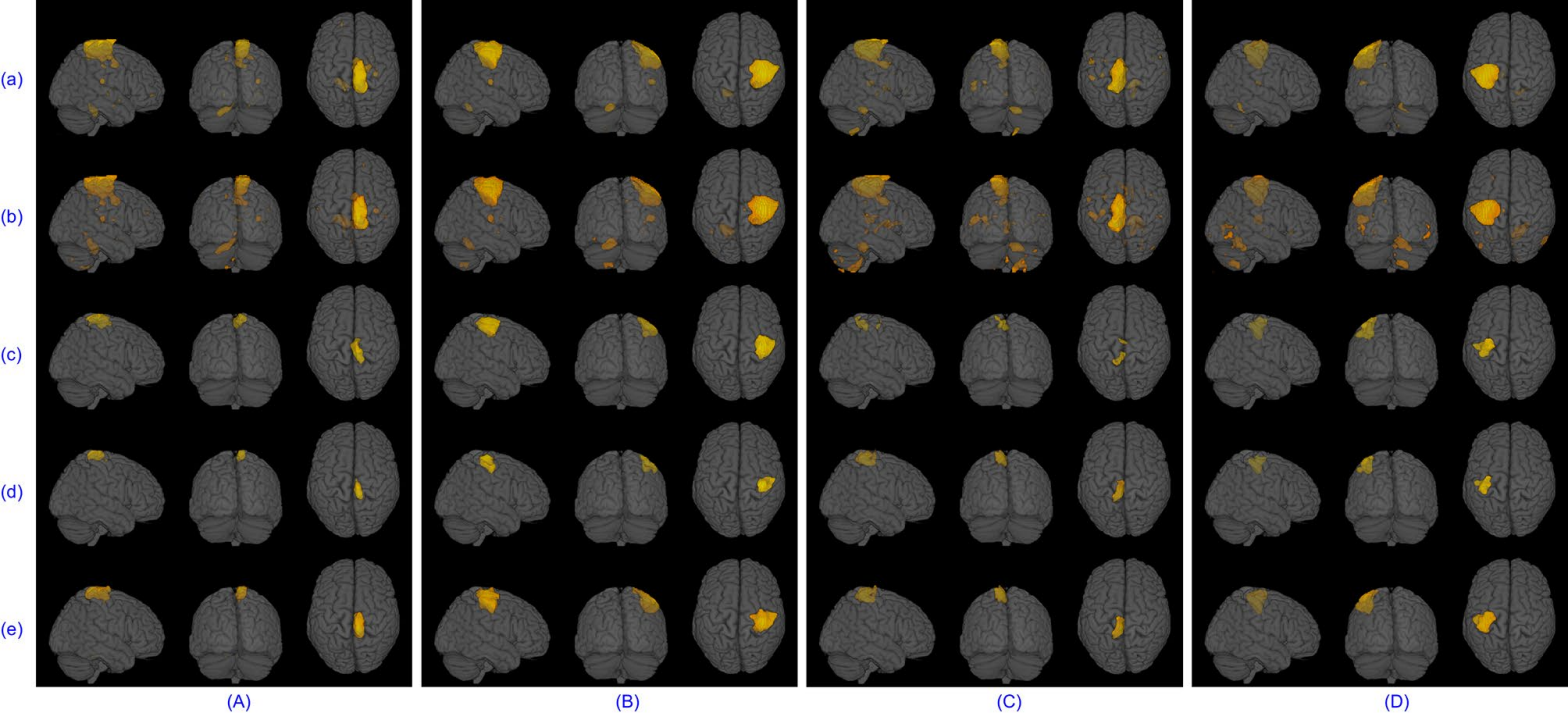
For the block design dataset’s left toe (**A**), left finger (**B**), right toe (**C**), and right finger (**D**) movement tasks, thresholded common activation maps at a random field correction $p<0.001$ obtained using (**a**) sgICA, (**b**) CODL, (**c**) ACSD, (**d**) swbDL, and (**e**) swsDL. The corresponding correlation values are given in Table 3.

**Figure 10:**
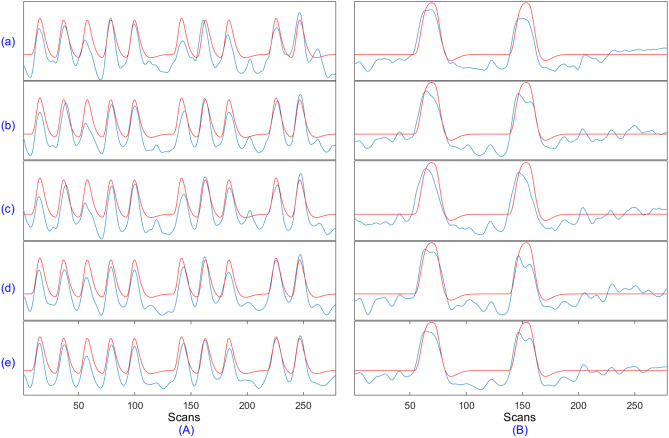
The most correlated group-level dictionary atom with MHR retrieved using (**a**) sgICA, (**b**) CODL, (**c**) ACSD, (**d**) swbDL, and (**e**) swsDL for the (**A**) visual cue, and (**B**) tongue movement task of the block design dataset. In Table 3, the corresponding correlation values are listed.

There were quite a few activation maps and temporal dynamics for the block design dataset, but only a few have been shown to avoid increasing the paper length. Spatial maps for left and right finger tapping tasks recovered by five algorithms for subject 4 are given in Fig. 8. A series of 2D images assembled to render 3D volume for left/right toe pinching and left/right finger tapping group-level tasks are shown in Fig. 9. The common temporal dynamics for visual cue and tongue and their MHR are plotted in Fig. 10. Tables 3 and 4 show that the proposed swsDL algorithm overall outperforms all other algorithms by yielding atoms/sparse codes having the highest correlation with the ground truth, while swbDL being the second-best for both group-level and individual analysis. From spatial maps in Figs. 8 and 9, it is pretty evident that the activations revealed by ACSD, swbDL, and swsDL are more specific to the motor area compared to the rest of the algorithms. Nevertheless, group-level maps revealed by swsDL are comparatively less specific and more sensitive.

**Figure 11:**
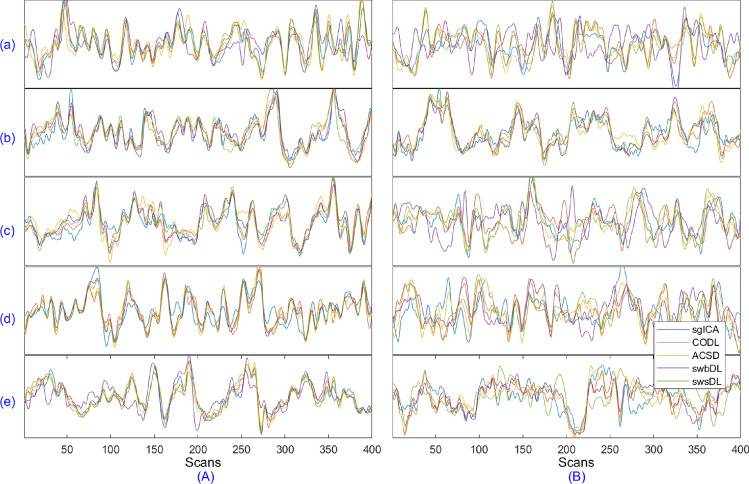
The TCs for subject number 1–5 shown in sub-figures a-e respectively extracted using sgICA, CODL, ACSD, swbDL, and swsDL for the (**A**) medial visual, and (**B**) frontoparietal left networks of the resting state dataset. Table 5 contains the corresponding correlation values and their means.

**Figure 12:**
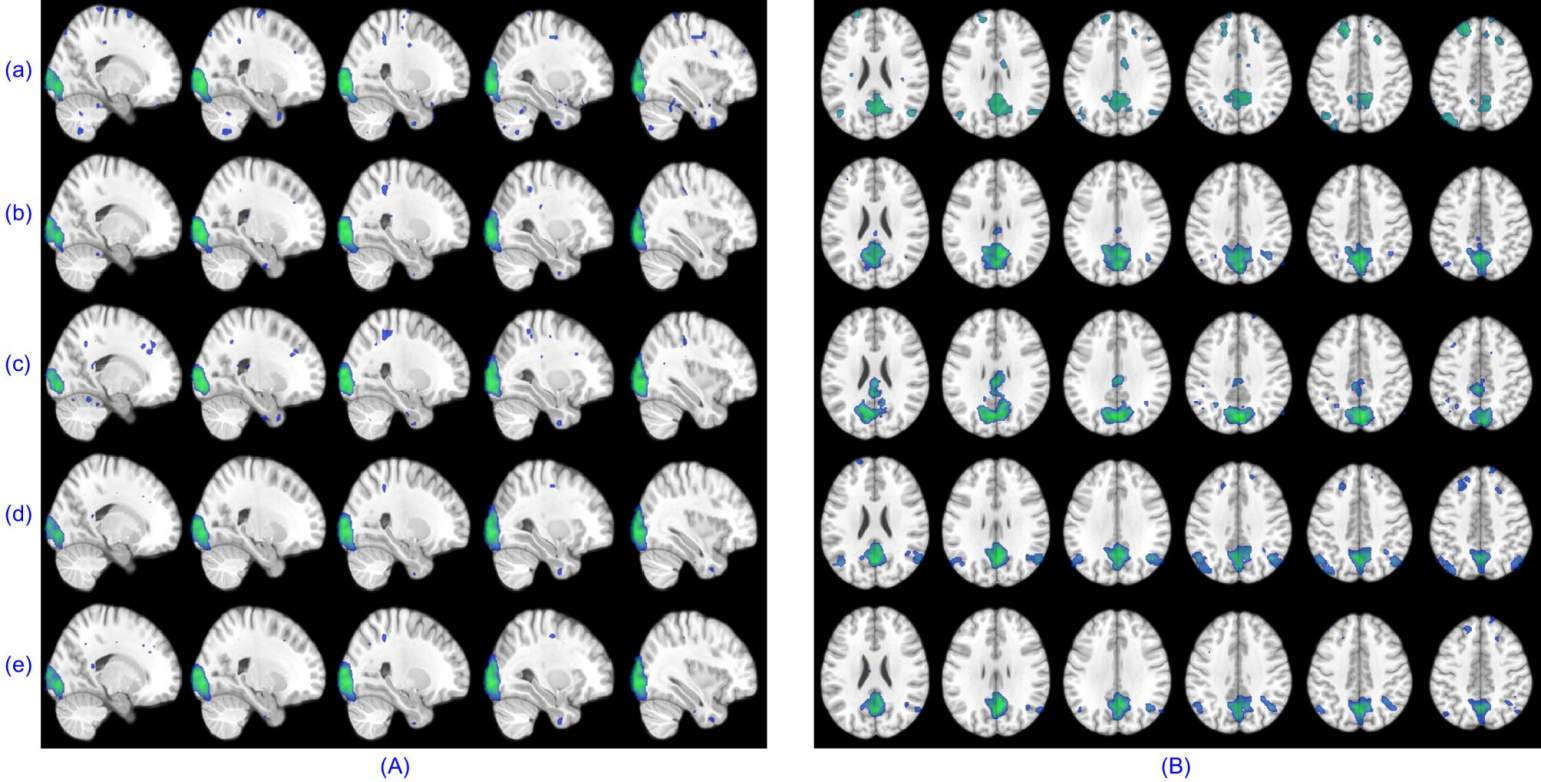
For the resting-state dataset’s RSN template 2 (**A**) and RSN template 4 (**B**), thresholded first subject’s activation maps at a random field correction $p<0.001$ obtained using (**a**) sgICA, (**b**) CODL, (**c**) ACSD, (**d**) swbDL, and (**e**) swsDL.

**Figure 13:**
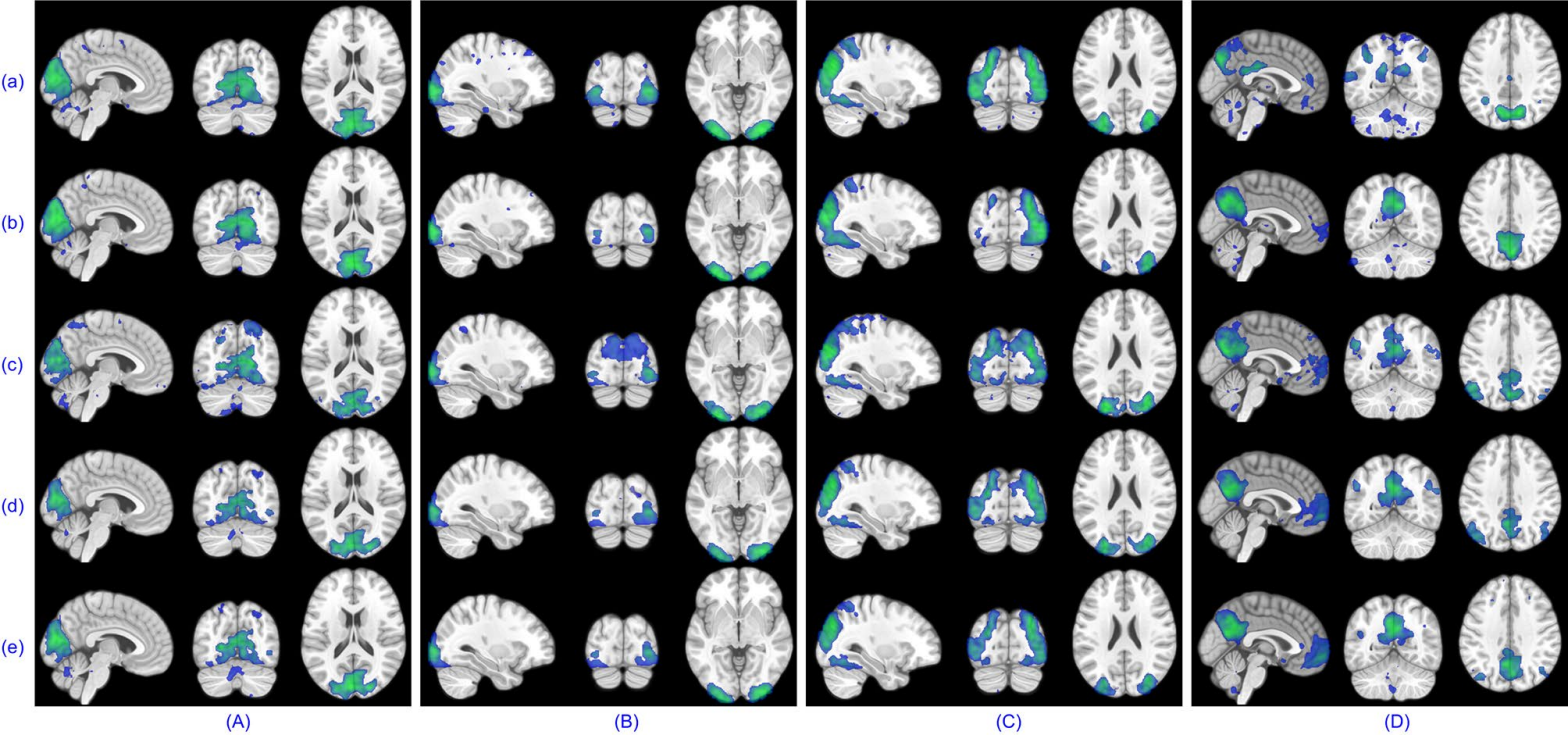
Thresholded group-level activation maps at a random field correction $p<0.001$ for the (**A**) RSN template 1, (**B**) RSN template 2, C) RSN template 3, and D) RSN template 4 obtained using (**a**) sgICA, (**b**) CODL, (**c**) ACSD, (**d**) swbDL, and (**e**) swsDL.

Only some results from the resting-state analysis have been shown here. For instance, (i) TCs obtained for the first five subjects using each of the five competing algorithms for medial visual and frontoparietal left network are shown in Fig. 11, (ii) the SMs for occipital pole visual and default mode network for subject 1 are shown in Figure 12, and (iii) group-level SMs for medial visual, occipital pole visual, lateral visual and default mode network are shown in Fig. 13. From Table 5, it can be concluded that overall, swsDL triumphed over all other algorithms in terms of correlation values. This is also visually supported by Figs. 12 and 13, where the spatial maps by swsDL appear more specific than maps by other algorithms.

**Figure 14:**
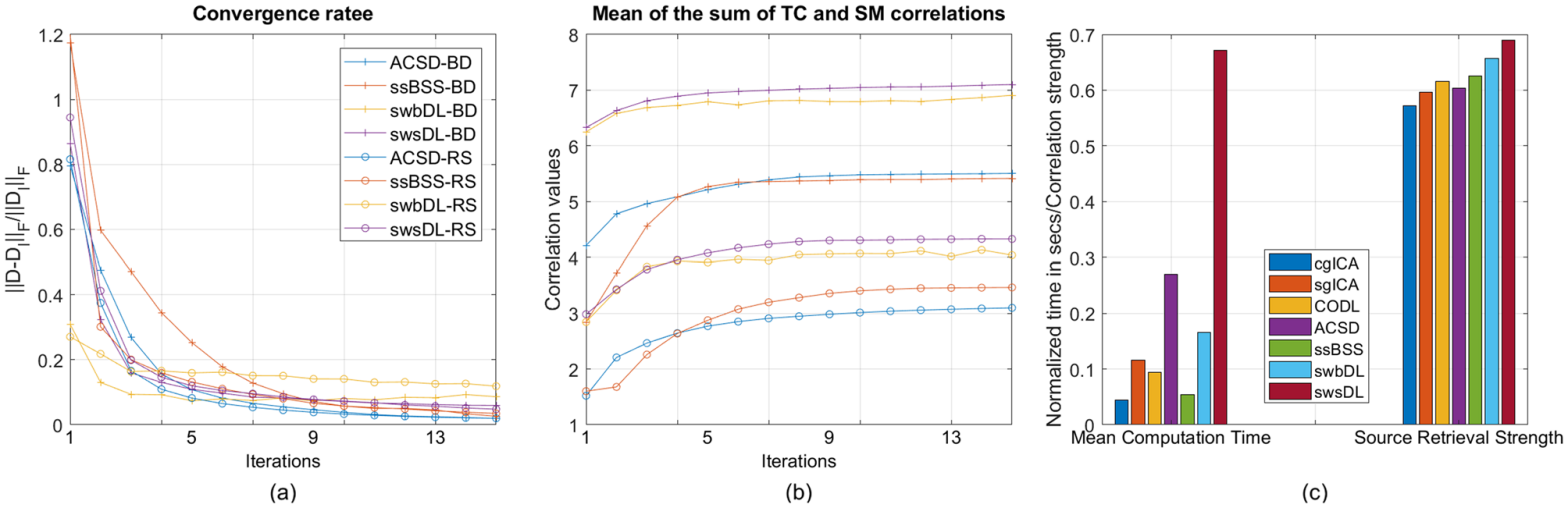
As a function of algorithm iterations, the mean of the (**a**) convergence rate and (**b**) correlation values over all subjects for subject-wise dictionary learning, and (**c**) time consumption and source retrieval strength by all competing algorithms.

For block design and resting-state datasets, Fig. 14 shows the convergence rate and correlation strength accumulation of ACSD, ssBSS, swbDL, and swsDL algorithms, and computational and source retrieval performance of all competing algorithms. The time values have been normalized to show correlation values and computation time in the same graph. It is also worth mentioning that the computation time is the mean of the time consumed by all three datasets, and the source recovery strength is the mean of subject-wise and group-wise correlation values for all three datasets.

Figure 14a shows that the ssBSS and ACSD algorithm and their proposed variants consistently converged over all iterations; however, this uniformity was less evident for swbDL. Figure 14b shows the steady development of correlation strength over all iterations for ssBSS, ACSD, and swsDL. In contrast, this progression seems to have stagnated for swbDL after the first few iterations. From these two subfigures, it becomes evident that compared to swbDL, both ACSD and swsDL gained from the increasing number of iterations. Figure 14c shows that although swsDL has outperformed all its predecessors, its computational performance is not very impressive. In contrast, swbDL has emerged as a runner-up in source recovery performance while having a relatively low numerical burden. This characteristic makes swbDL more favorable over swsDL/ACSD/ssBSS for FPGA-based implementation of DL algorithms in the future^70,71^.

## Conclusion

This paper has presented two new dictionary learning algorithms, swbDL, and swsDL, explicitly designed for subject-wise and group-wise analysis. Unlike the conventional group analysis, the proposed algorithms’ main advantage lies in their applicability to both task-related and resting-state fMRI data. Their efficacy has been illustrated using synthetic and experimental fMRI datasets, where their performance was found to be robust and unwavering across experiments. The computational simplicity of swbDL associated with lesser calls to data and lesser arithmetic operations makes it more favorable over all other DL algorithms. Both of the proposed algorithms are promising alternatives to ACSD, ShSSDL, and sgBACES algorithms. Reducing computational complexity associated with swsDL and its extension to hardware realization will be carried out in the future.

The strategy adopted for the proposed algorithms, where dictionary atoms and sparse codes are trained using the base spatiotemporal dynamics, is unprecedented. This approach allows incorporating similar components from the reduced-dimension space across subjects resulting in enhanced statistical power attained due to spatiotemporal variability offered by multi-subject data. The proposed model that considers this strategy through the training of representation matrices and base/dictionary sparse code is computationally intensive to solve. However, by exploiting the fast ssBSS method, block-wise update, and some performance compromise, a computationally efficient solution was reached via swbDL. On the other hand, an iterative approach was pursued using sequential learning that yielded higher source recovery precision at the cost of greater learning time. The convergence of both algorithms was guaranteed due to the sustenance of finite basis injective property and a strict sparsity pattern^72^.

## Data Availability

The experimental fMRI datasets used in this study are open-access and shared on the human connectome website. The Matlab code implemented for this study will be available from the corresponding author upon reasonable request.
